# MXene‐Integrated Printed Piezoresistive Flexible Sensors: A Breakthrough in Real‐Time Monitoring for Medical and Smart Applications

**DOI:** 10.1002/advs.202510894

**Published:** 2025-08-07

**Authors:** Hao‐wen Zhang, Xiang Xu, Di‐wen Jiang, Jie Lu, Yang‐sheng Wang, Zhe‐sheng Feng, Yan Wang

**Affiliations:** ^1^ School of Materials and Energy University of Electronic Science and Technology of China Chengdu 611731 China; ^2^ School of Materials Science and Engineering Zhejiang University Hangzhou 310027 China

**Keywords:** assisted deposition, machine learning, pressure sensor, stability, waffle‐structured MXene

## Abstract

As an emerging 2D transition metal carbide material with metal‐like conductivity, MXene exhibits significant potential in the development of piezoresistive sensors. Nevertheless, achieving favorable combined properties in MXene‐based pressure sensors remains challenging. This research explores a method for the assisted deposition of Ag nanoparticles between the layers of waffle‐structured MXene by template‐directed growth strategy, preparing the chocolate‐inlaid Ag@waffle‐structured MXene (WSM‐A8). With DFT calculations and finite element simulations, the theoretical analysis for the transition of energy bands and the increase of charge density is conducted for the WSM‐A8. Meanwhile, the conducting pathways established in WSM‐A8 are systematically simulated, verifying the construction of the oriented field‐modulation piezoresistive structure proposed in this study. The pressure sensor prepared with WSM‐A8 presents the highest Δ*I*/*I*_0_ response intensity (507 in 210 kPa) among the reported MXene‐based piezoresistive sensors with a satisfactory sensitivity (45/30 ms for response/recovery time), which also possesses outstanding structural stability (less than 4% attenuation of the response value after 500 bending cycles) and superior oxidation resistance. Utilizing the convolutional neural network and machine learning, the recognition accuracy of the integrated device is effectively improved. This study provides a feasible approach for realizing real‐time pressure monitoring, demonstrating great potential in medical diagnosis, intelligent actuators, and human–computer interactions.

## Graphical abstract

A chocolate‐inlaid Ag@waffle‐structured MXene is synthesized in this research via template‐directed growth strategy (WSM‐A8). The pressure sensor prepared with the WSM‐A8 presents the highest Δ*I*/*I*_0_ response intensity among the reported MXene‐based piezoresistive sensors with satisfactory sensitivity, outstanding structural stability, and superior oxidation resistance.

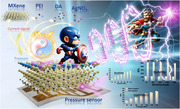

## 1 Introduction

As an essential component of wearable electronic sensing devices, flexible pressure sensors are highly demanded due to the rapid development of medical facilities, human–machine interaction, soft robotics, bionic electronic skin, and artificial intelligence.^[^ ^1^, ^2^, ^3^ ^]^ The manufacturing of real‐time pressure sensors with exceptional sensing properties, attenuation resistance, structural stability, and monitoring range is imperative.^[^ ^4^, ^5^ ^]^ With the advantages of metal‐like conductivity, ideal dispersibility, and excellent electronic response characteristics, materials such as graphene, carbon nanotube, conductive polymer, and boron‐nitrogen compound have been employed in the fabrication of pressure sensors with various response electrical signals in these years.^[^ ^6^, ^7^, ^8^, ^9^ ^]^ Nonetheless, due to the features of low response intensity and inferior sensitivity, the tendency to be easily oxidized, and the lack of anti‐deformation interference ability, the application scope of the pressure sensor that realizes the sensing function based on these materials is severely limited.^[^ ^10^, ^11^ ^]^ Meanwhile, the development of durable functional nanomaterial‐based flexible pressure sensors remains a challenge due to the restricted conductivity of the electrical signal conducting pathway during the process of compressive strain.^[^ ^12^, ^13^ ^]^ Therefore, it is significant to explore a high‐performance pressure sensing material with preferred sensitivity and prosperous physicochemical stability.

As an emerging 2D transition metal carbide material with a porous laminated structure and metal‐like conductivity, Ti_3_C_2_T*_X_* MXene has garnered extensive attention in the research of pressure sensitivity.^[^ ^14^ ^]^ The significant amount of transition metal elements and functional terminations that exist on the surface of MXene material endow it with the unique ability to transform the pressure‐induced structural strain into the conversion of conductivity, realizing the monitoring of the external pressure.^[^ ^15^, ^16^, ^17^ ^]^ However, with the increase of the applied pressure, the pressure sensitivity inevitably suffers from saturation due to the finite compressibility of the MXene material, which leads to the severe decline of the pressure monitoring property for the sensors.^[^ ^18^, ^19^ ^]^ Moreover, the inferior physical stability reduces the functional adaptability of the MXene‐based pressure sensor with different working environments in practical applications.^[^ ^20^, ^21^, ^22^ ^]^ Accordingly, it is of paramount importance yet highly challenging to bestow MXene material with exceptional structural stability, oxidation resistance, and favorable sensitivity performance.

To data, researchers have made numerous attempts to improve the comprehensive performance of the MXene‐based sensors in the aspect of pressure monitoring.^[^ ^23^, ^24^, ^25^, ^26^, ^27^, ^28^, ^29^, ^30^, ^31^, ^32^, ^33^ ^]^ Gao et al. have designed a skin‐like multifunctional sensor to precisely detect and distinguish the pressure stimuli without cross‐talk by decorating elastic and porous substrates with MXene nanosheets.^[^ ^24^ ^]^ Although the sensitivity of the reported sensor is respectable, the improvement in the response intensity and stability is still needed to adapt to the actual application. Based on the layered structure of the MXene, Lei et al. have realized the introduction of reduced graphene oxide particles, which effectively optimized the response intensity of the prepared piezoresistive functional sensor with the reduced graphene oxide as the rheostat between the layers of the MXene.^[^ ^25^ ^]^ However, the absence of antioxidant components in this design resulted in limited structural stability and oxidation resistance. Paolieri et al. have employed the 1–3, 4‐dihydroxyphenylalanine and polydopamine to modify the structural terminus of the MXene.^[^ ^26^ ^]^ The synergistic combination of polymer crosslinking and oxygen‐containing functional group incorporation substantially enhances the oxidative stability and structural integrity of MXene, establishing a novel dual‐functional modification strategy to improve the physical and chemical stability of MXene‐based sensing materials. Hence, despite conventional modification approaches often limited to unidimensional property enhancement, Ti_3_C_2_T*_X_* MXene demonstrates exceptional versatility. The MXene materials enable simultaneous optimization of piezoresistive sensitivity, mechanical durability, and environmental tolerance, thereby overcoming limitations in the development of pressure sensors.

Here, based on the waffle‐structured MXene (WSM) from the sensitization of polyethyleneimine (PEI) and dopamine (DA), we report a template‐directed growth strategy to construct chocolate‐inlaid Ag@waffle‐structured MXene (WSM‐A8) with structural stability, oxidation resistance, and exceptional pressure sensitivity for the preparation of the pressure sensor. The loading of the PEI/DA sensitization layer optimized the interlayer structure and activity of the obtained WSM, which is favorable for the assisted deposition of Ag nanoparticles and reinforcement of the physical stability. Meanwhile, the template‐directed growth of the Ag nanoparticles in the WSM has constructed an oriented field‐modulation piezoresistive structure for the composite material, which is beneficial for the construction of the electron transition network during the strain process of pressure monitoring. Moreover, the tight covering of the PEI/DA sensitization layer and oxygen‐containing groups generated during the sensitization process weakened the oxidation sensitivity of the WSM. Eventually, a real‐time pressure sensor with desirable pressure sensitivity and excellent physicochemical stability was prepared. Utilizing the prepared flexible pressure sensor, we have realized the recognition of the handwritten fonts. Meanwhile, we have assembled a wireless plantar pressure monitor for plantar pressure monitoring in challenging and demanding working environments. The accuracy of the recognition results for different Greek letters and standing postures reached 100% with the optimization of the machine learning algorithm. This research is of great significance for the monitoring of pulse rate, exercise state, information transmission, electronic skin, and wearable devices.

## 2 Results and Discussions

**Figure 1:**
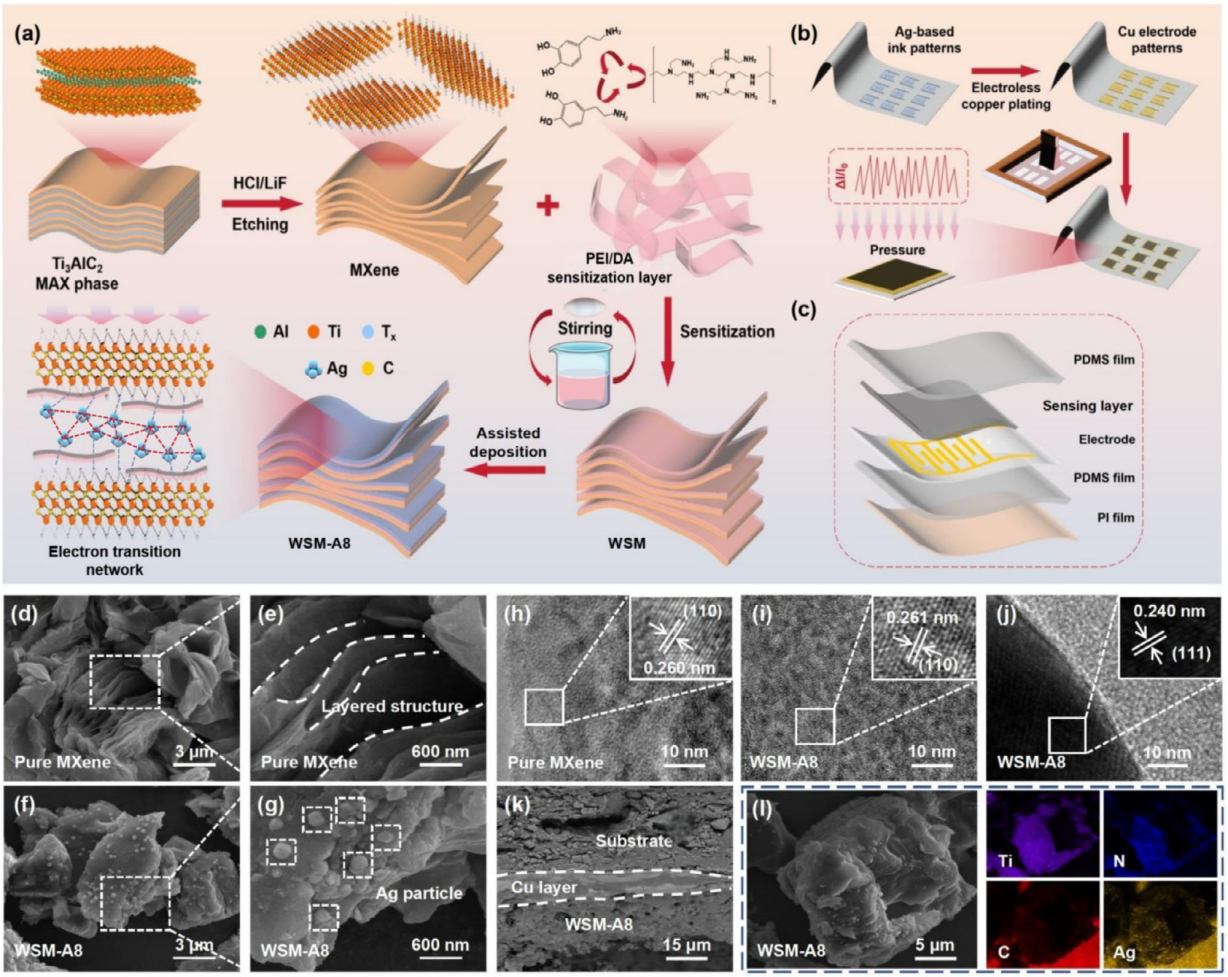
Fabrication process schematic of the WSM‐A8 pressure sensor. Preparation process of the a) chocolate‐inlaid Ag@waffle‐structured MXene and b) pressure sensing layer. c) Package structure of the WSM‐A8 pressure sensor. Low‐resolution SEM images of d) MXene nanosheets and f) WSM‐A8. High‐resolution SEM images of e) MXene nanosheets and g) WSM‐A8. h) High‐resolution TEM image of the MXene nanosheets. High‐resolution TEM image for i) MXene nanosheets and j) Ag nanoparticle of the WSM‐A8. k) Cross‐sectional SEM image of the pressure sensing layer. l) Elemental mapping image of WSM‐A8.

The preparation diagram for the WSM‐A8 is depicted in **Figure** **1**a, PEI and DA were employed to synthesize the sensitization layer on the MXene for the generation of the Ag nanoparticle and protection of the sensing material. In Figure 1b, the copper interdigital electrode was prepared through electroless copper plating (ECP), aiming to realize the large‐scale and highly efficient production. Figure 1c presents the package structure of the fabricated pressure sensor. A piece of polydimethylsiloxane (PDMS) film was covered on the pressure sensing layer for the conduction of the pressure variation signal. Another piece of PDMS film was pasted on the back surface of the paper‐based substrate to improve the flexibility and structural strength of the pressure sensor. Moreover, a piece of polyimide (PI) film was affixed on the bottom of the sensor to maintain the dryness of the sensor and mitigate the thermal effect of the operational environment. The surface morphology and microstructure of the sample were characterized by scanning electron microscopy (SEM) images and transmission electron microscope (TEM) images. As illustrated in Figure 1d, a laminated structure on the MXene sample was acquired through the etching of the MAX phase. During the etching process, the layered structure can be efficiently generated from the MAX phase with the exfoliation of the aluminum layer, which is similar to the morphology of the MXene sample formed by the etching of low‐concentration HF solution (Figure S1, Supporting Information). Furthermore, the opening layered structure in the high‐resolution SEM image further indicates the successful generation of the delaminated nanosheets (Figure 1e). Hence, it can be concluded that the MXene nanosheets with typical multilayered structures have been successfully prepared through this method. In Figure S2a (Supporting Information), the morphology of the WSM with sensitization layer is similar to the original MXene nanosheets. Meanwhile, the typical layered structure can be observed from the high‐resolution SEM image, demonstrating the structure stability of the MXene nanosheets during the sensitization treatment (Figure S2b, Supporting Information). Moreover, with the existence of the PEI/DA sensitization layer, Ag nanoparticles can be reduced from the AgNO_3_. As shown in Figure 1f, Ag nanoparticles are sufficiently covered on the surface of the WSM. The presence of the PEI/DA sensitization layer also promotes the template‐directed growth of the Ag nanoparticles on the interlayer of the WSM, which represents the successful formation of the WSM‐A8 (Figure 1g). On the other hand, almost all the MXene nanosheets in the low‐resolution SEM image possess a loose‐layered structure, demonstrating excellent repeatability of the preparation method proposed in this research (Figure S3a, Supporting Information). Also, distributed Ag nanoparticles can be investigated in different regions of the WSM‐A8, proving a high efficiency of the assisted deposition process (Figure S3b, Supporting Information).

As depicted in Figure 1h, a gap distance of 0.260 nm between the lattice fringes can be observed, corresponding to the (110) plane of the Ti_3_C_2_T_X_ MXene phase. Simultaneously, the lattice fringe for MXene nanosheets in Figure 1i exhibits a gap distance of 0.261 nm, corresponding to the (110) plane of the Ti_3_C_2_T_X_ MXene phase as well. Accordingly, no destruction for the crystal structure of the MXene nanosheets occurred during the preparation of the WSM‐A8. Synchronously, the gap distance of 0.240 nm between the lattice fringes corresponds to the (111) crystal plane of metallic silver, confirming the successful synthesis of the monoplasmatic Ag nanoparticles (Figure 1j). In addition, the wide distribution of the Ag nanoparticles with irregular spherical shapes and grain diameters approaching 250–300 nm in the low‐resolution TEM image also demonstrates the efficient template‐directed growth of silver nanoparticles (Figure S4, Supporting Information). As displayed in Figure S5 (Supporting Information), extra diffraction rings of (111) and (200) planes for the silver crystal can be identified in WSM‐A8, which also evidences the introduction of the Ag nanoparticles in the WSM‐A8. In Figure 1k, it is apparent that the interdigital electrode possesses a tight combination with paper‐based substrate and WSM‐A8. Meanwhile, the deposited copper layer as the interdigital electrode exhibits a consecutive morphology, high purity, and exceptional crystallinity, which is essential for the transmission of electric signals (Figure S6, Supporting Information). Additionally, as shown in Figure 1l, the elemental mapping images present the uniform distribution of Ti, N, C, and Ag elements on the pressure sensing layer, proving a satisfactory composition uniformity of the WSM‐A8.

**Figure 2:**
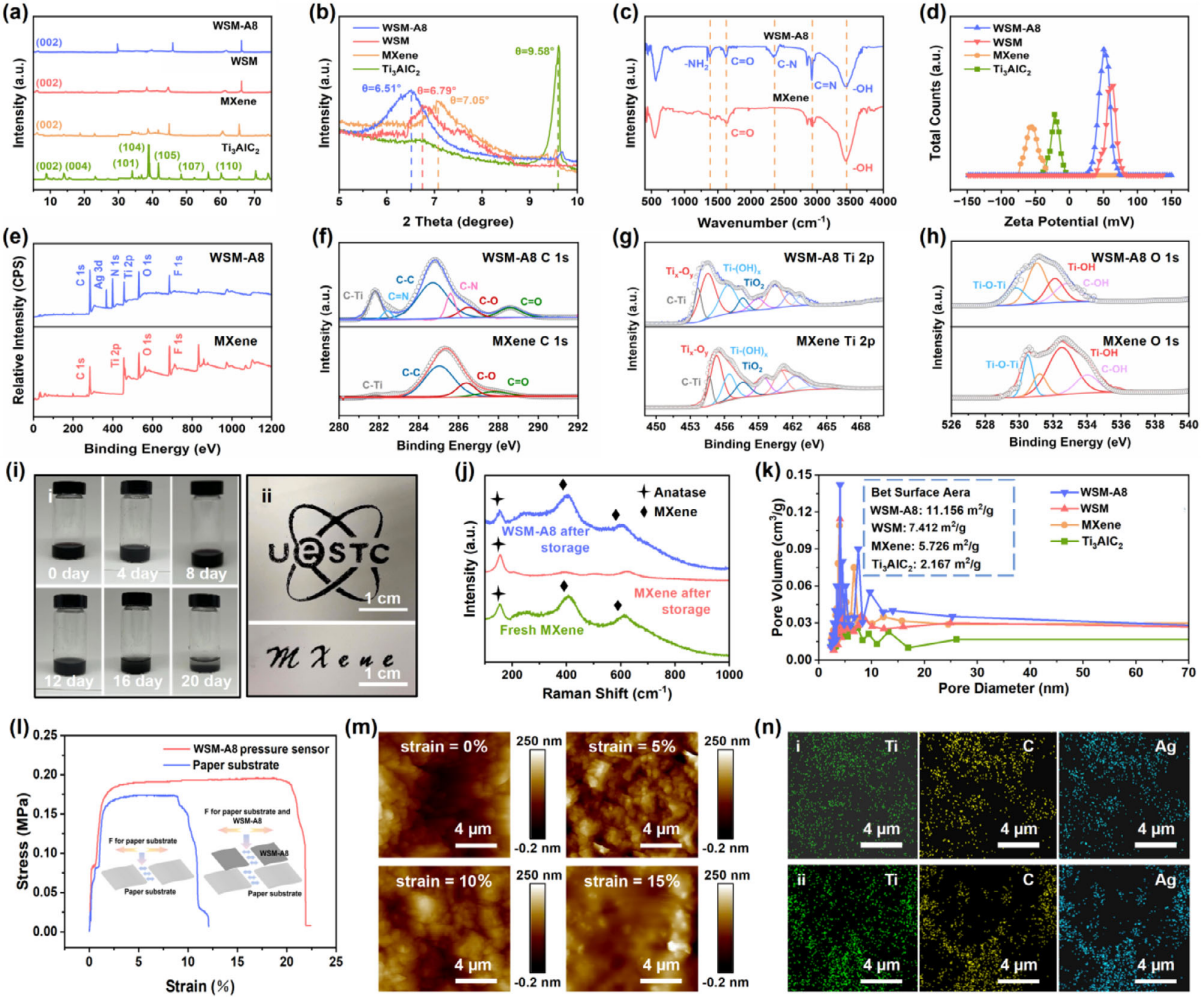
a) XRD patterns and b) low‐angle XRD patterns of Ti_3_AlC_2_ MAX phase, MXene nanosheets, WSM, and WSM‐A8. c) FT‐IR patterns of MXene nanosheets and WSM‐A8. d) Zeta potentials of Ti_3_AlC_2_ MAX phase, MXene nanosheets, WSM, and WSM‐A8. e) XPS survey spectrum of MXene nanosheets and WSM‐A8. XPS survey spectrum for f) C 1s, g) Ti 2p, and h) O 1s of MXene nanosheets and WSM‐A8. i) Photographs of i) WSM‐A8 after various days of storage, ii) printed WSM‐A8 patterns with different shapes. j) Raman spectra of the MXene, MXene after 10 d of storage, and WSM‐A8 after 10 d of storage. k) Pore size distributions of Ti_3_AlC_2_ MAX phase, MXene nanosheets, WSM, and WSM‐A8. l) Stress–strain curves of WSM‐A8 pressure sensor and paper substrate. m) AFM images of the copper electrode during the strain process. n) Elemental mapping image of the pressure sensing layer i) before and ii) after strain fracture.

As illustrated in **Figure** **2**a,b, X‐Ray diffraction (XRD) patterns and low‐angle XRD patterns are presented to investigate the crystal structures of Ti_3_AlC_2_ MAX phase, MXene nanosheets, WSM, and WSM‐A8, respectively. Compared to the Ti_3_AlC_2_ MAX phase, the diffraction peaks of MXene nanosheets, WSM, and WSM‐A8 decrease significantly at 39.2°, indicating the successful etching of the aluminum layer by Hydrochloric acid (HCl) and Lithium fluoride (LiF).^[^ ^34^, ^35^, ^36^, ^37^ ^]^ Besides, the Ti_3_AlC_2_ MAX phase contains a typical sharp peak center at 9.58°, assigned to the (002) crystalline plane. However, the (002) characteristic peak for MXene nanosheets, WSM, and WSM‐A8 exhibit a transition towards lower angles, broader shoulders, and lesser intensities. The results represent the layer spacing of the WSM‐A8 has been expanded to the maximum extent, confirming the successful loading of the Ag nanoparticles on the interlayer of the MXene nanosheets. As Fourier transform infrared spectrometer (FT‐IR) patterns depicted in Figure 2c, the peaks of MXene nanosheets at 3429 and 1638 cm^−1^ can be attributed to the tensile vibration of the ─OH and the stretching vibration of the C═O, corresponding to a typical chemical structure of the MXene. Moreover, the peak of WSM‐A8 at 1386 cm^−1^ corresponds to the symmetric stretching mode of the ─NH_2_, which is a representative functional group for PEI and DA. The peaks of WSM‐A8 at 2328 cm^−1^ and 2916 cm^−1^ are associated with the bending of the C─N and the vibration of the C═N, verifying the favorable combination of the MXene nanosheets with the PEI/DA sensitization layer. The tight combination between the MXene nanosheets and PEI/DA sensitization layer also effectively improved the adhesion strength of the subsequent template‐directed growth Ag nanoparticles. In Figure 2d, the zeta potentials are utilized to determine the magnitudes of surface charge for Ti_3_AlC_2_ MAX phase, MXene nanosheets, WSM, and WSM‐A8. The zeta potential of the MXene nanosheets was measured to be 21.3 mV lower than the Ti_3_AlC_2_ MAX phase, which is primarily induced by the negatively charged functional groups (halogenated hydrogen groups, hydroxyl groups, and carbonyl groups, etc.) introduced during the etching process. On the contrary, due to the existence of the positively charged functional groups on the PEI and DA, WSM and WSM‐A8 possess positive zeta potential values of 62.5 and 51.3 mV, which ulteriorly demonstrates the effective combination between the MXene nanosheets and PEI/DA sensitization layer.

The elemental composition and chemical bonding of the MXene nanosheets and WSM‐A8 are further investigated by X‐ray photoelectron spectroscopy (XPS) analysis. As shown in Figure 2e, the spectrum of WSM‐A8 exhibits an additional N 1s peak (399.4 eV) and Ag 3d peak (365.9 eV) compared to the MXene nanosheets, providing convincing evidence for the successful loading of the PEI/DA sensitization layer and Ag nanoparticles. In Figure 2f, four peaks are fitted in the C 1s spectrum of the MXene nanosheets at 281.4, 285.1, 286.6, and 288.1 eV, corresponding to C─Ti, C─C, C─O, and C═O respectively, which is consistent with the previously reported research, representing the typical chemical structure of MXene.^[^ ^38^, ^39^, ^40^, ^41^ ^]^ Additionally, two characteristic peaks at 282.6 eV and 285.4 eV corresponding to the C═N and C─N can be found in the C 1s spectrum of the WSM‐A8, resulting from the combination of the MXene nanosheets and the PEI/DA sensitization layer and the successful reaction between PEI and DA. As the thermal gravimetric analysis (TGA) curves of MXene nanosheets and WSM‐A8 illustrated in Figure S7 (Supporting Information), the WSM‐A8 reveals a weight‐loss ratio of 34.95% after deducting the weight‐loss ratio of MXene nanosheets, which is entirely from the thermal decomposition of DA and PEI. The differential TGA quantitatively confirms the high‐loading content of the PEI/DA sensitization layer in WSM‐A8. The Ti 2p spectrum displayed in Figure 2g reveals that the WSM‐A8 exhibits a more pronounced peak at 456.4 eV for Ti─(OH)*_x_* compared to MXene nanosheets, indicating the presence of ─OH groups introduced by DA. The Ag ions can be effectively reduced to Ag nanoparticles under the action of hydroxyl groups (The corresponding reaction mechanism can be found in Figure S8, Supporting Information). From the Ag 3d spectrum of the WSM‐A8, two strong peaks associated with Ag 3d_5/2_ and Ag 3d_3/2_ can be observed at 362.4 eV and 368.9 eV, which also demonstrates the formation of the Ag nanoparticles (Figure S9, Supporting Information). Furthermore, with the introduction of the oxygen‐containing groups, the oxidation resistance of the MXene nanosheets has been visibly improved (lower intensity of the TiO_2_ and Ti*_x_* ─O*_y_*). Due to the electron‐transfer ability of the hydroxyl groups, the progression of the oxidizing reaction can be effectively restrained. As the hydrogen ions in hydroxyl groups reacted with the oxyradical groups, the oxidation ability of oxidants can be effectively reduced through the transformation of electrons. As the O 1s spectrum is depicted in Figure 2h, the Ti─OH and C─OH located at 532.9 eV and 533.4 eV prove the existence of ─OH groups as well. At the same time, the Ti─OH peak is weakened and the C─OH peak is enhanced in the WSM‐A8 in comparison to MXene nanosheets, suggesting the hydrogen bonds are formed between the PEI/DA sensitization layer and MXene nanosheets. As a consequence, it can be summed that the PEI/DA sensitization layer has been successfully combined with the MXene nanosheets and the Ag nanoparticles have efficiently loaded on the interlayer of the MXene nanosheets.

As the photographs shown in Figure 2i, WSM‐A8 was stored for different numbers of days and printed with different shapes. After a long‐term storage period of 20 d, the WSM‐A8 began to appear deposition phenomena, indicating excellent storage stability. Moreover, WSM‐A8 can be transfer printed onto paper‐based substrates with different required shapes, exhibiting the pattern diversity and the favorable applicability for this material. To further characterize the chemical structure of the samples, the Raman spectra are indicated in Figure 2j. The spectroscopic analysis demonstrates that the spectrum of MXene nanosheets can be separated into bands of A_1g_ (Ti, C, O) and E_g_ (Ti, C, O) regions. The band at 300–470 cm^−1^ can be ascribed to the in‐plane (E_g_) vibrations of terminal groups bound with Ti atoms. The carbon vibrations including E_g_ and A_1g_ regions are allocated to the range between 580 cm^−1^ and 730 cm^−1^, revealing that the MXene nanosheets possess the chemical formula of Ti_3_C_2_F_2_, Ti_3_C_2_(OH)_2_, and Ti_3_C_2_O_2_. After 10 d of storage, the characteristic peak of MXene nanosheets can be found only at 157 cm^−1^, assigned to the E_g_ vibration of the anatase structure. Therefore, the material was completely converted to anatase TiO_2_ by oxidation. In contrast, the Raman spectrum exhibited by WSM‐A8 is almost identical to that of the initial MXene nanosheets, indicating excellent oxidation resistance for the prepared WSM‐A8. The pore size distributions of Ti_3_AlC_2_ MAX phase, MXene nanosheets, WSM, and WSM‐A8 are presented in Figure 2k. The pore volume of the WSM‐A8 is remarkably larger than that of WSM, MXene nanosheets, and Ti_3_AlC_2_ MAX phase. Meanwhile, the specific surface area of the WSM‐A8 (11.156 m^2^ g^−1^) is found to be 50.51% higher than that of the WSM (7.412 m^2^ g^−1^), 94.83% higher than that of the MXene nanosheets (5.726 m^2^ g^−1^), and 414.81% higher than that of the bulk MAX phase (2.167 m^2^ g^−1^). Hence, it can be concluded that the structure of the MXene nanosheets has been optimized to a larger specific surface area through the intercalated loading of Ag nanoparticles, thereby enhancing the potential of the compressive strain for WSM‐A8 and promoting pressure sensitivity. In addition, under the same condition, the stronger nitrogen adsorption capacity for WSM‐A8 further confirms our conjecture (Figure S10, Supporting Information). In Figure 2l, with the WSM‐A8 as the pressure sensing layer covered on the paper substrate, the ductility of the pressure sensor can be effectively improved compared to the paper substrate due to the polymer properties of the WSM‐A8 during the tensile strain process (the rise speed of the tensile stress was 2 N s^−1^, and the decline speed was 5 N s^−1^). Besides, under the plastic deformation regime after prolonged strain processing, the surface roughness of the copper electrode has almost no change (Figure 2m). Therefore, the pressure sensing layer can be firmly attached to the electrode, guaranteeing the stable transmission of electrical signals. The elemental mapping images in Figure 2n proved the uniformity of the pressure sensing layer attached to the substrate before and after the strain fracture, which also demonstrates the stability of the pressure sensing layer.

**Figure 3:**
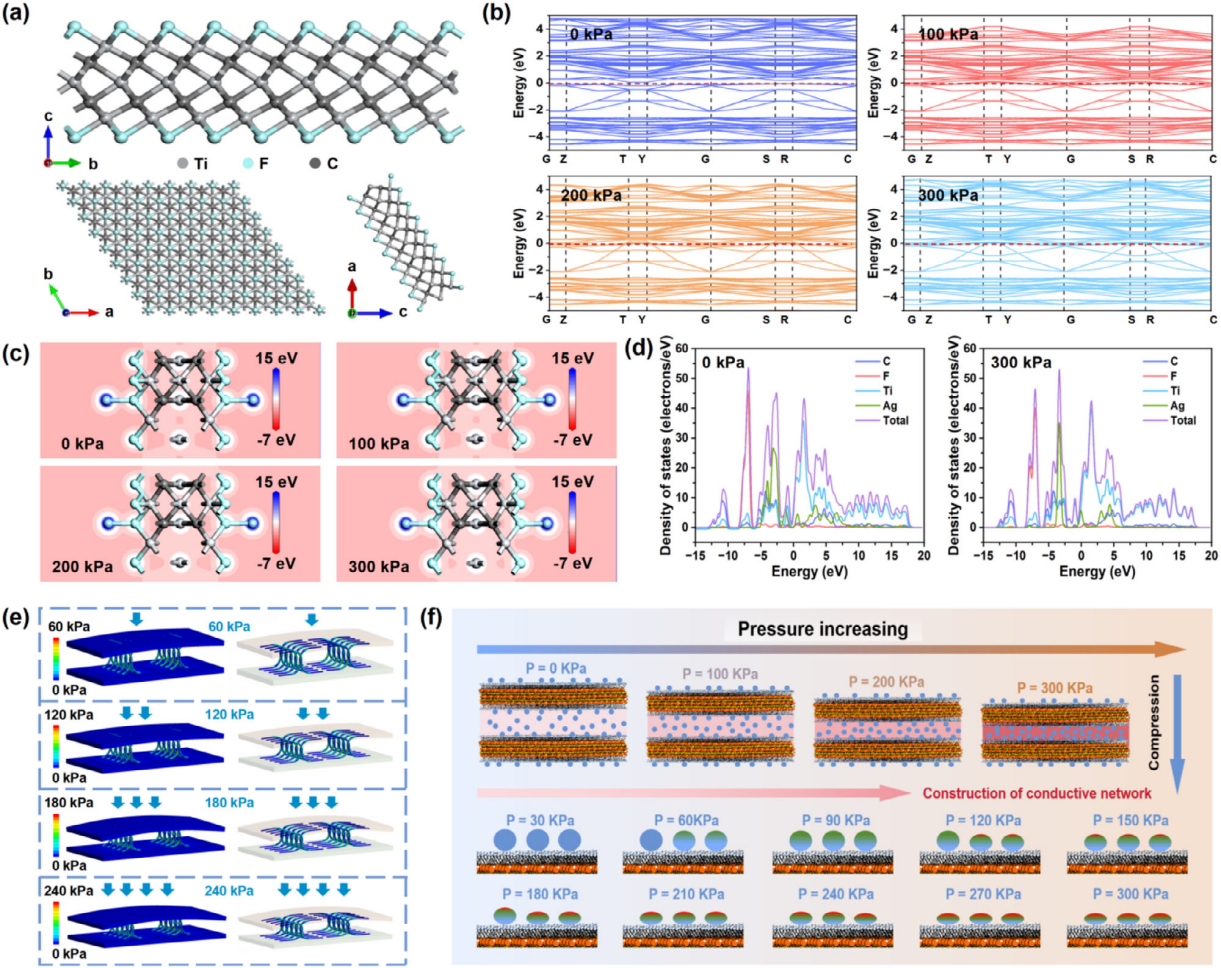
a) Atomic structure of the MXene viewed in different directions. b) Energy band structures of the MXene under different pressure conditions. c) Charge density diagrams of the WSM‐A8 under different pressure conditions. d) The density of states for the WSM‐A8 under different pressure conditions. e) Finite element analysis of the stress distribution for WSM‐A8 under different pressure conditions. f) Pressure sensing mechanism of the WSM‐A8 pressure sensor.

In order to characterize the electronic structure and the sensing mechanism of the WSM‐A8, density functional theory (DFT) calculations were performed. **Figure** **3**a illustrates the structure of the MXene material for the first‐principles calculations. Under different pressure conditions, pressure stimuli promote the transition of the partial energy bands, indicating the potential of MXene‐based materials in the application of the pressure sensing material (Figure 3b). Meanwhile, as shown in Figure 3c, the higher pressure conditions lead to the increase of the charge density for the WSM‐A8, which proves that more electrons can be transferred between the electron transition network. In Figure 3d, compared with the WSM‐A8 under the condition of no pressure applied, the electronic density of the states surrounding the Fermi level overlaps more under the pressure condition of 300 kPa for WSM‐A8. The results suggest that the WSM‐A8 has better charge transfer and conductivity characteristics under higher pressure conditions, which matches well with our theoretical speculation. Furthermore, the intrinsic 2D layered architecture of MXene enables anisotropic piezoresistive modulation in WSM‐A8, wherein externally applied pressure along the monitoring axis dynamically regulates interlayer charge polarization through strain‐mediated lattice deformation. As the finite element analysis displayed in Figure 3e, the WSM‐A8 would be deformed with the pressure applied, creating more conducting pathways in the network and resulting in a change in resistance, which is helpful for the interpretation of the sensing mechanism for the WSM‐A8 pressure sensor. On the other hand, the structure stability of the MXene materials can be effectively ensured under different pressure conditions, presenting the function stability of the WSM‐A8 pressure sensor during the monitoring process.

Under the constant supply voltage, the sensing mechanism of the WSM‐A8 pressure sensor in this research is revealed in Figure 3f. The sensing ability of the flexible piezoresistive pressure sensor is determined by the adjustable variations in the conductive network under external pressure. With the increased monitored pressure on the sensor plane, the WSM‐A8 is compressed and the contact area between the adjacent 2D conductive MXene nanosheets is elevated, creating a more conducting electron transition network. Therefore, the overall resistance of the WSM‐A8 pressure sensor is significantly decreased. Additionally, the incorporation of chocolate‐inlaid Ag nanoparticles expands the interlayer spacing of WSM‐A8, significantly enhancing its potential for resistance variation and compressive deformation, thereby improving the overall sensing performance of the pressure sensor. Also, in the process of pressure monitoring, the reduction of the distance between the chocolate‐inlaid Ag nanoparticles and the compressive strain of the structure for chocolate‐inlaid Ag nanoparticles promotes the elevation of the on‐off ratio for the conductive network. Meanwhile, the densely distributed chocolate‐inlaid Ag makes the construction of the conductive network more sensitive, effectively improving the minimum detectable limit of the WSM‐A8 pressure sensor. On the other hand, with a continuous increase in the loading pressure, the sensitivity of the WSM‐A8 pressure sensor displays a gradual decrease and approaches saturation subsequently, which is ascribed to the limitation of the elastic deformation. Therefore, the WSM‐A8 pressure sensor proposed in this research possesses an excellent monitoring scope with exceptional sensitivity. The preceding sensing mechanism matches with the subsequent experimental results well.

**Figure 4:**
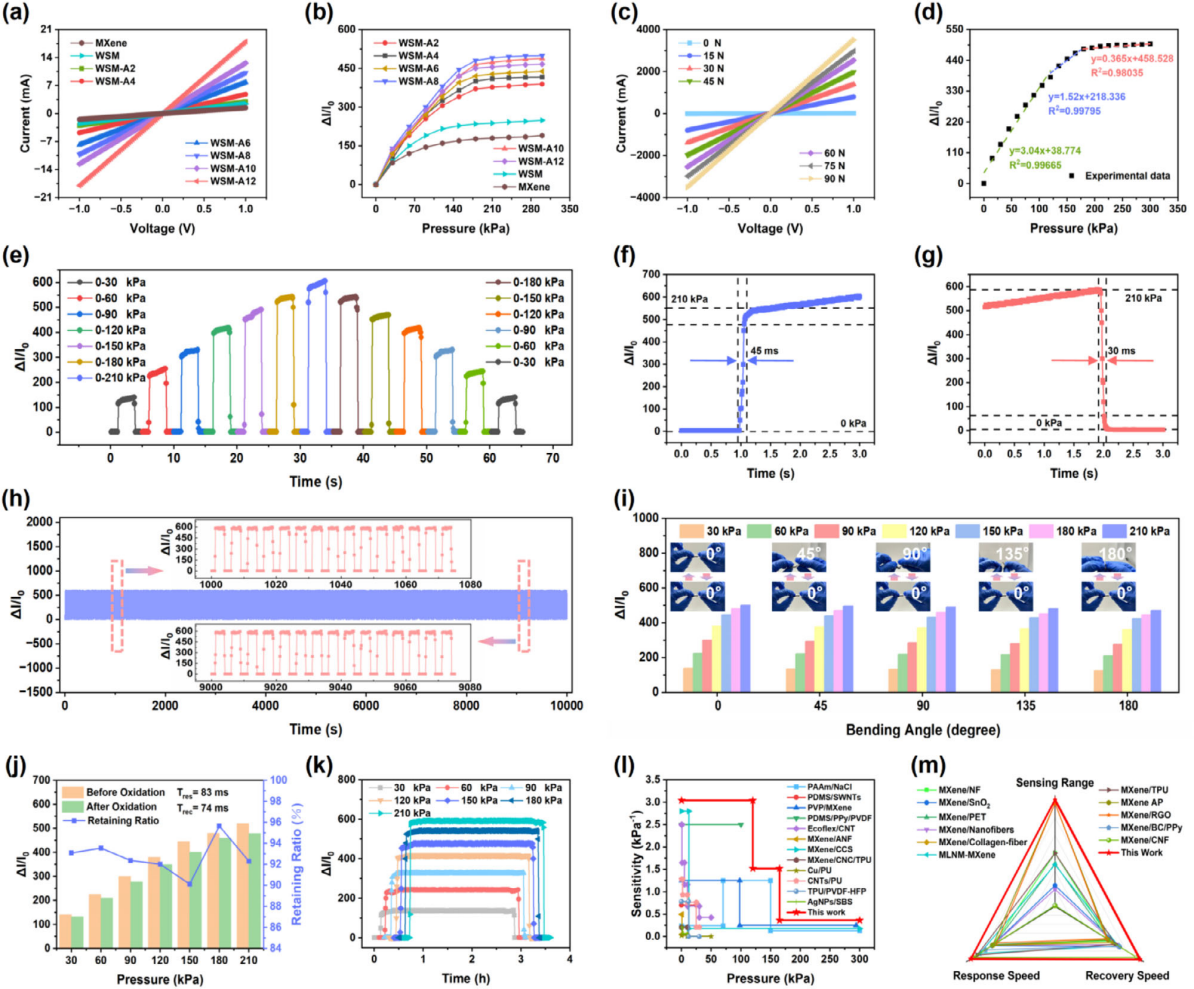
a) *I–V* curves of the WSM‐Aa, WSM, and MXene pressure sensors. b) Response curves of the WSM‐Aa, WSM, and MXene pressure sensors under different pressure conditions. c) *I–V* curves of the WSM‐A8 pressure sensor under the various static pressures. d) Sensitivity of the WSM‐A8 pressure sensor. e) Dynamic response and recovery characteristics of the WSM‐A8 pressure sensor. f) T_res_ and g) *T*_rec_ for WSM‐A8 pressure sensor. h) Cycle stability test of the WSM‐A8 pressure sensor. i) Response variations of the WSM‐A8 pressure sensor after 500 bending cycles. j) Variation of response for the WSM‐A8 after 20 d of exposure to air. k) Response stability of the WSM‐A8 pressure sensor. l) Comparison of the monitoring sensitivity, m) response/recovery sensitivity, and sensing range for the WSM‐A8 pressure sensor with other pressure sensors.

The *I–V c*urves of the WSM‐Aa (a represents the concentrations of AgNO_3_ during the preparation of the material, which has been explained in Experimental Section) pressure sensors were obtained under the condition of dynamic voltage (**Figure** **4**a). The electric conductivity of the WSM‐Aa pressure sensor increased with the concentration increase of AgNO_3_, demonstrating that the assisted deposition of the Ag nanoparticles can be effectively promoted with a higher concentration of AgNO_3_ during the preparation of the WSM‐Aa. As presented in Figure 4b, the WSM‐A8 pressure sensor exhibits the highest Δ*I*/*I*_0_ values under the different pressure conditions among the WSM‐Aa pressure sensor. It can be found from the comparison of the results, when the concentration of the AgNO_3_ exceeds 8 mg mL^−1^ during the preparation process, the excessive accumulation of the Ag nanoparticles on the interlayer of the composites may compromise the interfacial stability and restrict the deformation potential of the sensing layer, leading to the decline of the response intensity and sensitivity of the sensor. Meanwhile, when the concentration of the AgNO_3_ is below 8 mg mL^−1^ during the preparation process, the electric conductivity for the sensing layer of the pressure sensor is too low to transmit the electrical signals generated during the sensing process effectively, thereby compromising signal integrity and response/recovery speed. Hence, the concentration of AgNO_3_ at 8 mg mL^−1^ represents the best experimental approach. Based on the relationships between the thickness of WSM‐A8 and Δ*I*/*I*_0_ values, it can be found that the response intensity of the pressure sensor declined slightly with the increase of WSM‐A8 thickness. (Figure S11,Supporting Information). Therefore, 90 µm is the optimum thickness of the pressure sensing layer in this research. Figure 4c shows the *I‐–V* curves of the WSM‐A8 pressure sensor under various static pressures. The reverse currents have the same characteristics and strict linear behaviors as the forward currents for the WSM‐A8 pressure sensor, implying an excellent ohmic contact between the pressure sensing layer and copper electrodes. Hence, the operating voltage has no effect on the response characteristics of the sensor. In addition, the response intensity of current increases obviously, revealing positively correlated compatibility with the increment of loading pressure. As the fitting curves of the Δ*I*/*I*_0_ values illustrated in Figure 4d, the sensitivity parameter for the WSM‐A8 pressure sensor can be equated as 3.04, 1.52, and 0.365 kPa^−1^ at the regions of 0–120, 120–180, and 180–300 kPa, respectively. Hereby, the WSM‐A8 pressure sensor is endowed with great potential in practical applications due to the wide sensing range and high sensitivity. The dynamic response and recovery curves of the WSM‐A8 pressure sensor are displayed in Figure 4e. With the variations of pressure conditions, the Δ*I*/*I*_0_ values of the WSM‐A8 pressure sensor change significantly between the 0 kPa and various pressure conditions (matches well with the *I–V* curves), confirming an ultra‐high response intensity and favorable ability to distinguish various levels of stress accurately. Moreover, a pressure stimulus of 210 kPa was applied to detect the response time (*T*_res_) and recovery time (*T*_rec_) of the WSM‐A8 pressure sensor. Maintaining low numerical values for *T*_res_ and *T*_rec_ serves as the fundamental basis for real‐time monitoring of pressure. In general, *T*_res_ and *T*_rec_ are measured as the time required for the pressure sensor to reach 90% of the equilibrium value. As shown in Figure 4f,g, the *T*_res_ and *T*_rec_ of the WSM‐A8 pressure sensor are 45 and 30 ms, respectively, suggesting the highest sensitivity among the reported MXene‐based pressure sensors and facilitating real‐time monitoring during the application process.

The WSM‐A8 pressure sensor entails encountering various intricate scenarios during the application process, necessitating a comprehensive durability evaluation. After repeated compression for 2000 cycles under the pressure of 0–210 kPa, the dynamic response curves for the WSM‐A8 pressure sensor remain a high degree of coincidence with the initial test results owing to the satisfactory resilience of the WSM‐A8 and PDMS substrate, validating excellent cycling stability for the WSM‐A8 pressure sensor (Figure 4h). Furthermore, bending tests have been conducted to verify the structural stability of the WSM‐A8 pressure sensor. With 500 cycles of bending at the angles of 45°, 90°, 135°, and 180°, it can be found in Figure 4i that the Δ*I*/*I*_0_ values of the WSM‐A8 pressure sensor declined slightly. Meanwhile, the descent degree of the ΔI/I_0_ values is enhanced as the increase of bending angle. Nevertheless, the decrease ratio of the Δ*I*/*I*_0_ value is less than 4% at the bending angle of 180° indicating an outstanding structural stability for the WSM‐A8 pressure sensor. In addition to the physical stability, chemical stability is also an indispensable property. When exposed to air, MXene materials are susceptible to oxidation. As presented in Figure 4j, after the storage, pressure sensor was prepared with the stored WSM‐A8 and the oxidation resistance of the WSM‐A8 was demonstrated by comparing the Δ*I*/*I*_0_ values of the prepared sensor. The Δ*I*/*I*_0_ values of the WSM‐A8 pressure sensor in different pressure conditions are reduced to a certain extent after the WSM‐A8 being stored for 20 d. In this process, the Δ*I*/*I*_0_ values under the 150 kPa reveal a maximum decrease ratio of 9.12% and under the 180 kPa reveal a minimal decrease ratio of 3.78%. Thus, the decrease ratio of the Δ*I*/*I*_0_ values for the WSM‐A8 pressure sensor in different pressure conditions is less than 10%, which are in an ideal range. Besides, the *T*_res_ and *T*_rec_ have slightly increased to 38 and 44 ms, respectively. Hence, the real‐time transmission of the response signal is almost unaffected. Based on the above results, it can be concluded that the WSM‐A8 pressure sensor possesses a superior oxidation resistance. Additionally, the high latency of the signal‐receiving terminal is also a common problem during the operation process of electronic equipment. As depicted in Figure 4k, the response signal for the WSM‐A8 pressure sensor can be maintained stably for a long period (over 3 h), which is essential for the identifiability of the signal‐receiving terminal to the response signal. Finally, the monitoring sensitivity, response/recovery sensitivity, and sensing range of the WSM‐A8 pressure sensor have been compared with the previously reported pressure sensors. In Figure 4l and Table S1 (Supporting Information), the WSM‐A8 pressure sensor exhibits an apparently higher monitoring sensitivity in each sensing range, which is caused by the unique layered structure and the Ag nanoparticles that exist on the interlayer of the WSM. At the same time, in comparison with other typical MXene‐based pressure sensors, the WSM‐A8 pressure sensor displays a faster response and recovery speed with a wider sensing range (Figure 4m and Table S2, Supporting Information). In conclusion, the WSM‐A8 pressure sensor demonstrates excellent physicochemical stability and satisfactory capabilities for real‐time pressure monitoring, possessing significant potential for practical applications.

The pulse rate and radial artery augmentation index (ratio between the heights of the systolic peak and diastolic peak in a pulse waveform, *r*_AI_ = *P*_2_/*P*_1_) serve as crucial indicators of real‐time physiological information in the human body, playing a pivotal role in the prevention and diagnosis of numerous diseases. As shown in Figure S12 (Supporting Information), the WSM‐A8 pressure sensor has enormous application value in real‐time pulse monitoring and timely emergency response. Besides, the interference caused by the wrist bending has no effect on the original pulse waveform and pulse rate, presenting a great wrist posture fault tolerance of the WSM‐A8 pressure sensor for real‐time pulse monitoring (Figure S13, Supporting Information). During the process of exercise, prolonged keeping in an incorrect posture is considered to be a tremendous inducing factor for diseases including osteoarthritis and varicose veins. In Figure S14 (Supporting Information), with WSM‐A8 pressure sensor during exercise is beneficial for the adjustment of exercise posture and the prevention of diseases. On the other hand, from the perspective of electronic communication, information security has become a significant issue that cannot be ignored. The WSM‐A8 pressure sensor can be utilized to convey information using the dots and dashes of Morse code. This communication method plays an irreplaceable role in the field of emergency rescue when a disaster strikes (Figure S15, Supporting Information). Furthermore, the WSM‐A8 pressure sensor can be affixed to the throat to capture the vocal cord vibrations for different word pronunciations, which is constructive to enhance the development of the encryption and transmission of security information (Figure S16, Supporting Information).

**Figure 5:**
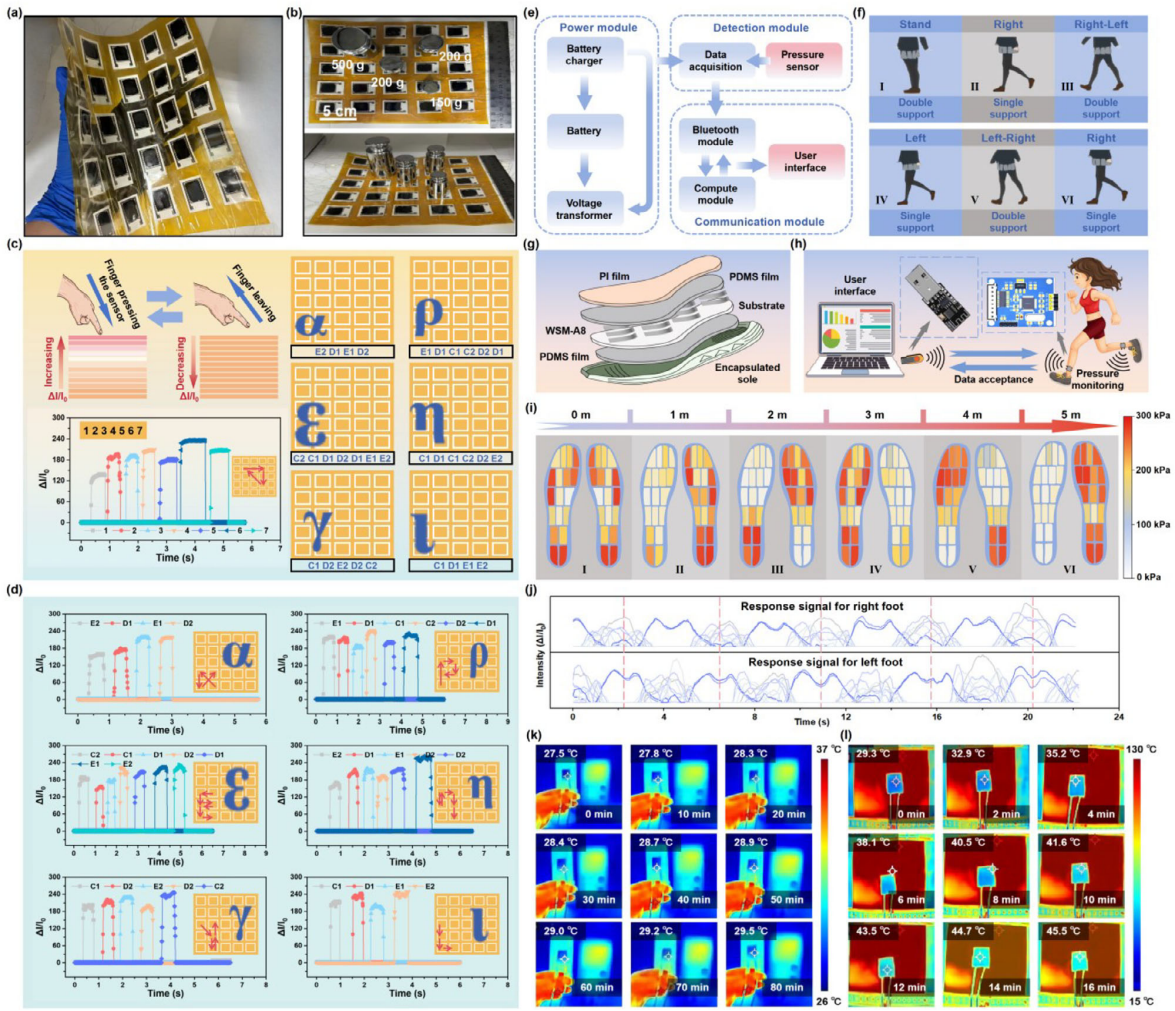
a) Photographs of 5 × 5 WSM‐A8 pressure sensor array. b) Photographs of weights with different qualities placed on the sensor array. c) Schematic of the finger inducing the response signal by pressing the sensor array and the recognition sequence for the handwritten Greek letters. d) Schematic of the recognition for the handwritten Greek letters with the corresponding response signal. e) Schematic for the functional modules of the wireless WSM‐A8 plantar pressure monitor. f) Schematic of the plantar pressure monitoring during the major monitoring phases of a full gait cycle. g) Perspective view layout of the wireless WSM‐A8 plantar pressure monitor. h) Schematic of the working principle for the plantar pressure monitor. i) Plantar pressure monitoring results. j) Response signal from 8 typical integrated sensors on the tow feet during the plantar pressure monitoring. k) Infrared thermal images of the WSM‐A8 pressure sensor with a supply voltage of 3 V. l) Infrared thermal images of the WSM‐A8 pressure sensor on the heating platform.

To prove the concept for the electronic skin, the WSM‐A8 pressure sensors with a size of 25 mm × 18 mm were assembled into a flexible sensor array with 5 × 5 pixels (**Figure** **5**a). Concurrently, in order to verify the real‐time pressure monitoring of different regions on the sensor array, weights with qualities of 500, 200, and 150 g are placed on the sensor array (Figure 5b). As depicted in Figure S17 (Supporting Information), the flexible sensor array is able to identify the magnitudes and positions of the applied pressure accurately. Additionally, the flexible sensor array can be attached to the surface of the wearable fabric, which is employed to achieve real‐time pressure monitoring of the human body (Figure S18a, Supporting Information). With a palm pressing on the surface of the sensor array, the pressure exerted on the human body in various areas can be precisely monitored (Figure S18b, Supporting Information), revealing a prospective development potential in the realm of safety protection and human–computer interaction. As a further initiative, six adjacent pixels in the array are chosen to design an intelligent input terminal, aiming to recognize handwritten Greek letters (Figure S19, Supporting Information). As the finger presses on the different pixels, the response signals within the corresponding channels can be recorded (Figure 5c and Figure S20, Supporting Information). Based on the sensor pixels preassigned on the array, six common Greek letters can be recognized through the triggering of the sensor pixel in a certain order, which also provides an innovative development strategy for the HMI systems (Figure 5d). Moreover, with the change of the applied pressure on the pressure sensor, the brightness of the LED light in the circuit elevates rapidly due to the improvement of the current response, displaying the capabilities of the WSM‐A8 pressure sensor in the domains of stress alert and circuit protection (Figure S21, Supporting Information).

In Figure S22 (Supporting Information), a wireless communication‐based WSM‐A8 plantar pressure monitor was assembled for plantar pressure monitoring in challenging and demanding working environments. The corresponding functional modules are illustrated in Figure 5e, which mainly consists of the power module, detection module, and communication module. With the wireless WSM‐A8 plantar pressure monitor, the plantar pressure distribution during various phases of static standing and dynamic walking in humans can be effectively monitored (Figure 5f). During the process of practical application, the WSM‐A8 plantar pressure monitor can be wrapped in an encapsulated sole, which would provide comprehensive protection for it (Figure 5g). Meanwhile, this protective structure has no effect on the data transmission at all (Figure 5h). The pressure mapping diagrams displayed in Figure 5i accurately depict the plantar pressure distributions of different regions for the tester. It can be found that specific areas for the sole of the foot (such as the heel area and anterior metatarsal area) ordinarily bear greater pressure during the process of walking, which should be provided with better safeguards in particular operational environments. Meanwhile, combined with the detailed continuous electrical output signal, this technology is profitable for the evaluation of plantar health status and rehabilitation training of disease (Figure 5j and Figure S23, Supporting Information). Overall, the WSM‐A8 plantar pressure monitor assembled in this research can be utilized for professionals in various fields, including sports science, rehabilitation engineering, and biomechanics. Eventually, as shown in Figure 5k and Figure S24 (Supporting Information), the temperature increase rate of the WSM‐A8 pressure sensor is extremely slow with a constant supply voltage of 3 V due to the sluggish generation of joule heating, which is profitable to reduce the thermal effect and prolong the equipment lifespan. In addition, placing the WSM‐A8 pressure sensor on the heating platform with an initial temperature of 130 °C to characterize the thermal insulation property of the pressure sensor with the infrared thermal images (Figure 5l). It can be observed that after a period of 16 min, the temperature of the upper surface for the pressure sensor increases from 29.3 °C to 45.5 °C merely, indicating a favorable thermal insulation property. At the same time, the response intensity of the WSM‐A8 maintains desirable stability during this process (Figure S25, Supporting Information). Consequently, the structure designed with PDMS film and PI film provides protection for the WSM‐A8 pressure sensor in the actual working environment.

**Figure 6:**
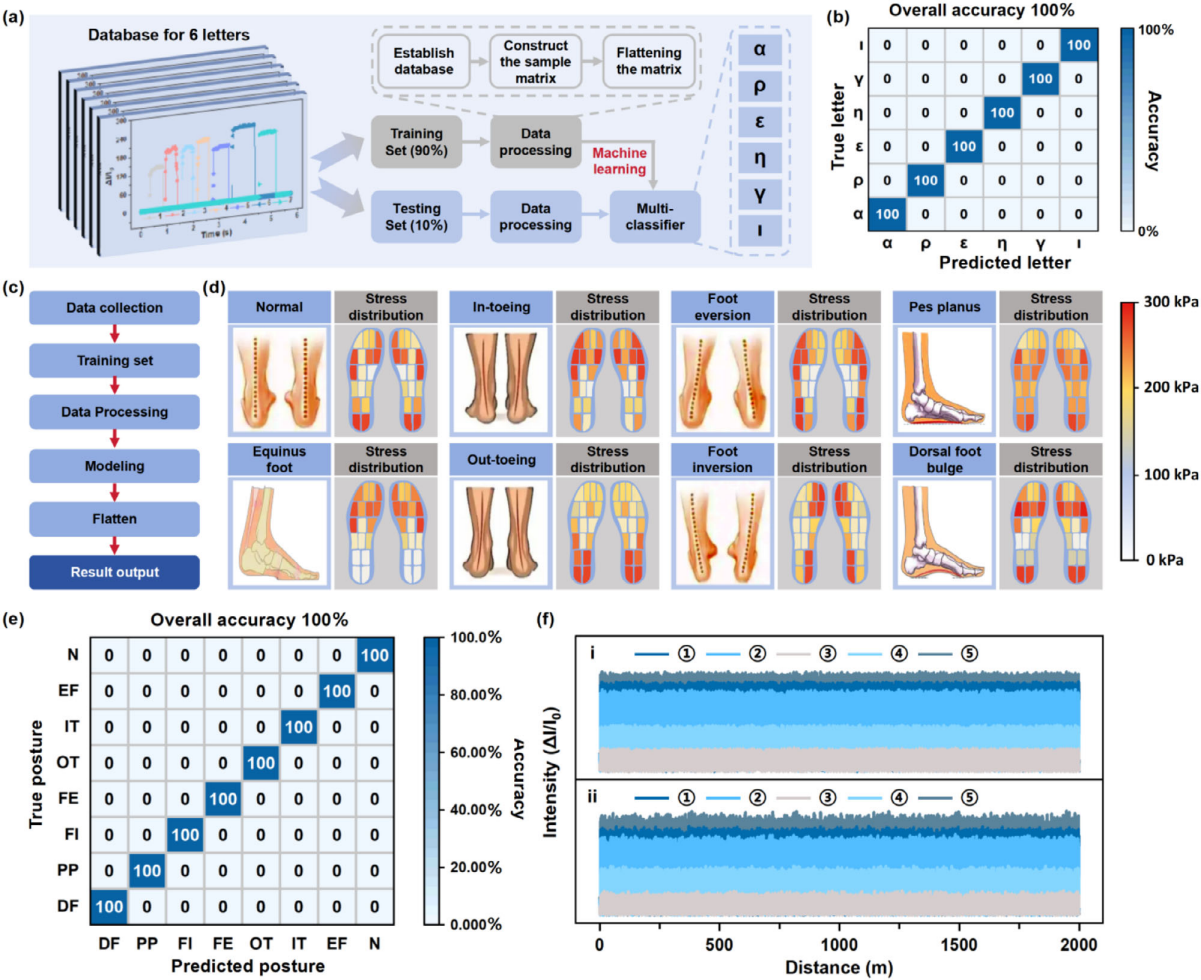
a) Flow chart of the machine learning algorithm for Greek letter recognition. b) Confusion map for the recognition results of different Greek letters. c) The object recognition process in machine learning for WSM‐A8 plantar pressure monitor. d) Different types of plantar pressure distributions matched with the standing postures. e) Confusion map for the recognition results of different standing postures. f) Response signal for the 5 selected points in the WSM‐A8 plantar pressure monitor from the i) left foot and ii) right foot during walking for 2 km.

To improve the recognition accuracy of Greek letters in human‐computer interaction, a machine learning algorithm based on convolutional neural networks (CNN) has been designed to classify and identify the data obtained from the sensor array. To build the database, the voltage signal corresponding to each letter was tested 1000 times with the data used for training and testing in a ratio of 4:1 to realize the accurate recognition (**Figure** **6**a). According the training and validating by CNN classification algorithm, resulting in a recognition accuracy of 100% for the 6 different Greek letters (Figure 6b). Similarly, various plantar pressure distributions lead by the different standing postures also can be classified and identified by CNN classification algorithm (Figure 6c). As depicted in Figure 6d, 8 types of plantar pressure distributions were captured, matching to the 8 different standing postures (The standing postures include the normal (N), equinus foot (ET), in‐toeing (IT), out‐toeing (OT), foot eversion (FE), foot inversion (FI), pes planus (PP), and dorsal foot bulge (DF)). For the building of the database, 800 plantar pressure distribution data for each foot were collected for the training and 200 plantar pressure distribution data were collected for the testing. Impressively, the recognition accuracy for different standing postures reached 100% after the testing (Figure 6e). The accuracy can be primarily attributed to the precise plantar pressure distributions captured by the WSM‐A8 plantar pressure monitor, suggesting a promising future for integrating machine system recognition with plantar pressure sensing systems. To verify the property stability of the WSM‐A8 plantar pressure monitor under long‐term working conditions, 5 representative stress points on the plantar have been selected and the response signal has been observed (Figure S26, Supporting Information). As the response signal curves displayed in Figure 6f, the response signal of the monitor exhibits stable monitoring characteristics after 2 km of exercise, presenting great potential for long‐term monitoring of the WSM‐A8 plantar pressure monitor. In conclusion, the WSM‐A8 pressure sensor can be considered a promising candidate in electronic skin applications, showing a promising potential for the next‐generation wearable sensor.

## 3 Conclusion

In summary, this work demonstrates a piezoresistive printed flexible sensor with excellent pressure sensitivity and physicochemical stability, enabling facile, durable, and stable real‐time monitoring of the piezoresistive signal. Based on the template‐directed growth strategy, the WSM‐A8 with oriented field‐modulation piezoresistive structure was prepared to be employed as the functional sensing material. The generated Ag nanoparticles in the WSM‐A8 constructed an oriented field‐modulation piezoresistive structure for the composite material, facilitating the construction of the electron transition network during the pressure monitoring. DFT calculation and finite element analysis have been employed for theoretical analyzing the construction of the oriented field‐modulation piezoresistive structure. Due to the satisfactory piezoresistive property of the sensing material, the prepared printed flexible sensor presents high sensitivity within the 0–210 kPa detection range with exceptional stability, enduring over 2000 cycles of periodic testing at 210 kPa and resisting fatigue under complex stress conditions. By combining with machine learning, these advancements facilitate high‐precision intelligent recognition of plantar types and biomedical applications, enhancing the capabilities of wearable devices for health detection, human‐computer interaction, and medical rehabilitation.

## 4 Experimental Section

### Materials

HCl was purchased from Acmec Biochemical Co., Ltd. (Shanghai, China). LiF was purchased from Xilong Scientific Co., Ltd. (Chengdu, China). Ti_3_AlC_2_ MAX phase was purchased from Kelong Chemical Co., Ltd. (Chengdu, China). DA and AgNO_3_ were purchased from Oddo's Biological Technology Co., Ltd. (Nanjing, China). PEI (M. W. 10000) was purchased from 11 Technology Co., Ltd. (Jilin, China). All reagents adopted in the experiments were not further purified.

### Synthesis of the MXene Nanosheets

To prepare the MXene nanosheets, 1.5 g LiF powder was poured into 30 mL of 10 m HCl solution at a polytetrafluoroethylene lined reactor and stirred for 40 min at 35 °C. With the complete dissolution of LiF powder, 1.5 g Ti_3_AlC_2_ MAX phase was gradually added into the above dispersing fluid. The obtained mixed solution was subsequently stirred at 45 °C for 26 h constantly. After the reaction, the resulting suspension was washed with deionized water and centrifuged (4500 rpm) for 8 min recurrently until the pH approached about 7. Finally, fully drying the precipitate in a vacuum oven to acquire the MXene nanosheets.

### Preparation of Chocolate‐Inlaid Ag@waffle‐Structured MXene

To prepare the PEI/DA sensitization solution, 4 g DA powder and 2.5 g PEI were mixed into 30 mL deionized water in a polytetrafluoroethylene container with ammonia solution to adjust the pH of the solution approached to approximately 7. Based on the gentler ultrasonic waves provided by the bath ultrasonication than probe‐style ultrasonication, the mixed solution was placed into a bath ultrasonication system maintained at 70 °C for 1 h. After the ultrasonic treatment, the obtained solution was stirred at 60 °C for 2 h to finish the preparation of the PEI/DA sensitization solution. Then, 2 mL PEI/DA sensitization solution was intermingled with 0.2 g MXene nanosheets and ultrasonic treatment for 2 h. After the ultrasonic treatment, the solution was transferred into a reactor vessel and subjected to hydrothermal reaction for 2 h at 85 °C to generate the WSM. Ultimately, various quality of AgNO_3_ powder was blended with the WSM and subjected to continuous stirring at 70 °C for a duration of 2 h to realize the template‐directed growth of the Ag nanoparticles and complete the preparation of chocolate‐inlaid WSM‐Aa, where a represented the concentrations of AgNO_3_ (2, 4, 6, 8, 10, and 12 mg mL^−1^).

### Fabrication of the Pressure Sensor

The high‐quality copper interdigital electrode was prepared through ECP according to the previous reports.^[^ ^42^, ^43^ ^]^ First, the special silver‐based ink was transfer‐printed onto the paper‐based substrate through the flexible plate printing equipment to pattern the interdigital electrode catalytic site. After that, the printed pattern was exposed to the UV device to be solidified with a wavelength of 400–460 nm. Subsequently, the solidified substrate was immersed in an ECP solution to generate the copper layer and form the interdigital electrode. The schematic of the copper electrode is depicted in Figure S27 (Supporting Information). Next step, the WSM‐A8 was directly transfer‐printed onto the interdigital electrode through a mesh filter with square patterns, aiming to form the pressure sensing layer. Eventually, the printed pressure sensing layer was fully dried in a vacuum oven at 55 °C for 12 h.

### Characterization and Measurement

The surface morphology of the sample was observed using JSM‐6490LV scanning electron microscope (JEOL, Japan). The microstructure of the sample was investigated by JEM 2100F transmission electron microscope (JEOL, Japan). The crystal structure of the sample was performed by XRD‐7000 X‐ray diffraction analyzer at a scan rate of 20° min^−1^ in Cu Kα radiation (*λ* = 1.5418 Å) (Shimadzu, Japan). The spectral variation of the sample was obtained by Nicolet‐IS5 FT‐IR spectrometer (Thermo Scientific, USA). Under the vacuum pressure lower than 10^−2^ Torr, the specific surface and pore size distribution of the sample were analyzed using Kubo‐X1000 surface analyzer via the Brunauer‐Emmett‐Teller test method with the degassing process conducted at 110 °C for 5 h (Builder, China). The chemical bonding structure of the sample was characterized by Kratos XSAM800 X‐ray photoelectron spectroscopy system (Kratos, UK). The thermogravimetric analysis of the sample was determined by HS‐TGA‐101 thermal gravimetric analyzer (Hesheng instrument, China). The zeta potential of the sample was tested by ZS90 Zeta potential analyzer (Malvern, UK).

The uniform compressive force was provided by ZQ‐990B universal testing machine (Zhiqu, China). The current response of the sample was acquired with Th2830 capacitance resistance analyzer (Thonghui, China). The testing voltage for the analyzer is 1 V and the work frequency for the analyzer is 200 Hz. The experimental facility for the pressure sensing test is displayed in Figure S28 (Supporting Information). The current–voltage curve of the sample was measured by CS350H electrochemical workstation (Junhuiteng, China). The relative change in current (Δ*I*/*I*_0_) is defined as the response signal of the pressure sensor and reaction for the magnitude of the sensitivity. The corresponding calculation equation is defined as Δ*I*/*I*_0_ = (*I* – *I*_0_)/*I*_0_, where *I* is the instantaneous current and *I*_0_ is the initial current. Besides, sensitivity is also an important parameter for evaluating the sensing performance of the pressure sensor, which is defined as the ratio of the relative change in current and pressure. The corresponding calculation equation for the sensitivity parameter of the pressure sensor (*S*) is defined as *S* = (Δ*I*/*I*_0_)/Δ*P*, where Δ*P* is the relative change in pressure. All the tests were conducted at room temperature (25 °C). Details of finite element analysis, DFT calculation and machine learning are described in the Supporting Information.

### Statistical Analysis

All quantitative data were calculated as the mean values derived from triplicate experimental measurements to ensure statistical reliability and minimize variability. The measurement results for sensing property were visualized using linear graphical representations to systematically elucidate the dynamic response. Each dynamic sensing property was tested three times to reduce the measurement errors and enhance repeatability in empirical studies. The statistical analysis was conducted with Origin 8.0 (Origin Lab, USA).

## Conflict of Interest

The authors declare no conflict of interest.

## Supporting information



Supporting Information

## Data Availability

The data that support the findings of this study are available from the corresponding author upon reasonable request.
