# Supplementary material for: MXene‐Integrated Printed Piezoresistive Flexible Sensors: A Breakthrough in Real‐Time Monitoring for Medical and Smart Applications
Source: Adv Sci (Weinh). 2025 Aug 7;12(40):e10894. doi: 10.1002/advs.202510894 (PMC12561432; doi:10.1002/advs.202510894)
Supplement: Supplementary file 1 — Supporting Information [file ADVS-12-e10894-s001.docx]

**Supporting Information**

**MXene-integrated Printed Piezoresistive Flexible Sensors: A Breakthrough in Real-time Monitoring for Medical and Smart Applications**

Hao-wen Zhang, Xiang Xu, Di-wen Jiang, Jie Lu, Yang-sheng Wang, Zhe-sheng Feng*, and Yan Wang*

Dr. H. w. Zhang, D. w. Jiang, J. Lu, Dr. Y. s. Wang, Prof. Z. s. Feng, Prof. Y. Wang

School of Materials and Energy, University of Electronic Science and Technology of China, Chengdu 611731, China

E-mail: wy@uestc.edu.cn

Dr. X. Xu

School of Materials Science and Engineering, Zhejiang University, Hangzhou 310027, China


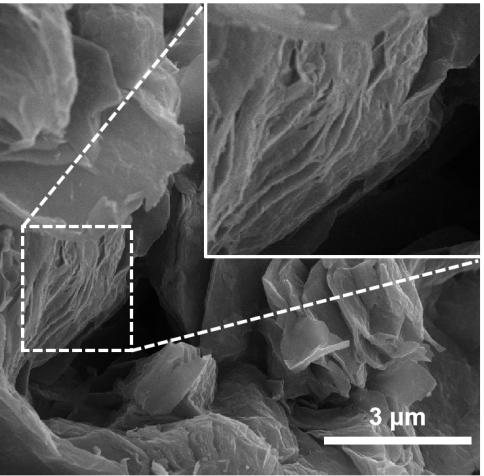


**Figure S1.** SEM image of the MXene nanosheets prepared by etching of low-concentration HF solution.


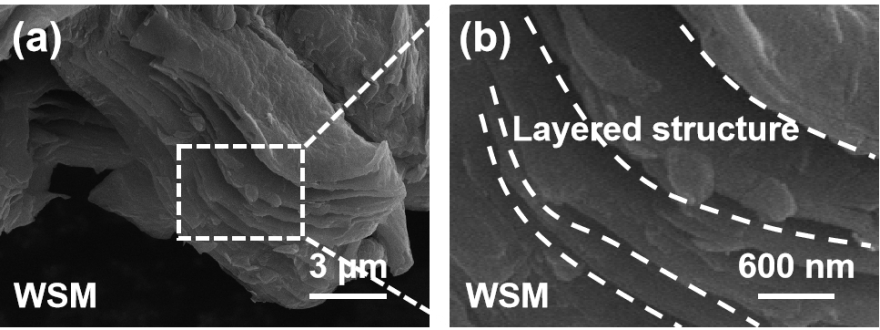


**Figure S2.** (a) Low-resolution SEM image and (b) High-resolution SEM image of the WSM.


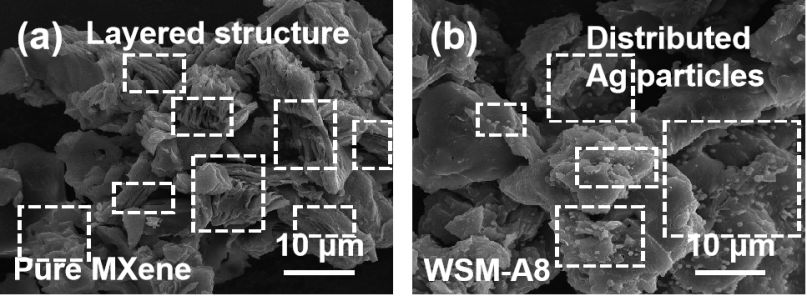


**Figure S3.** (a) Low-resolution SEM image of the distributed MXene nanosheets. (b) Low-resolution SEM image of the distributed Ag nanoparticles.


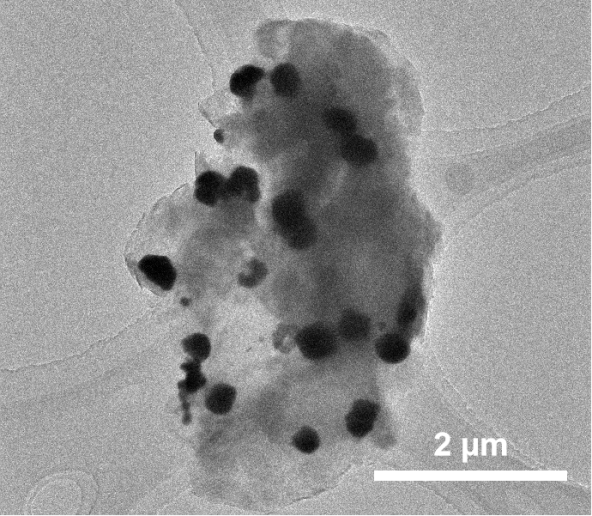


**Figure S4.** Low-resolution TEM image for MXene nanosheets and Ag nanoparticle of the WSM-A8.


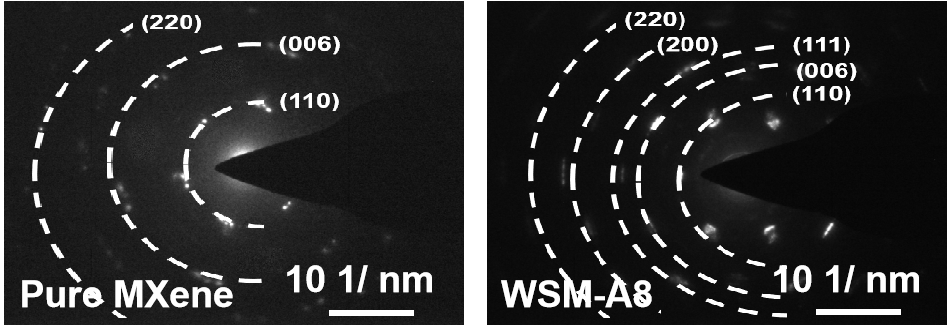


**Figure S5.** SAED patterns of MXene nanosheets and WSM-A8.


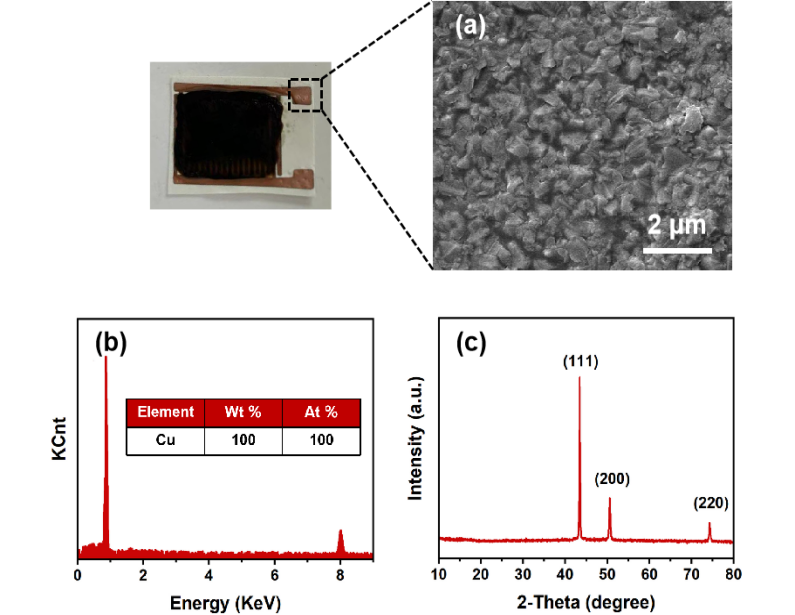


**Figure S6.** (a) SEM image of the copper layer prepared by electroless copper plating. (b) EDS of the prepared copper layer. (c) XRD patterns of the prepared copper layer.


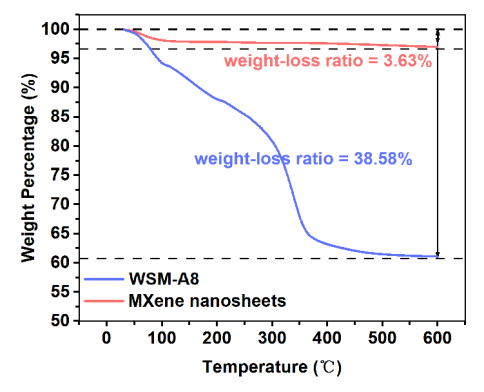


**Figure S7.** TGA curves of MXene nanosheets and WSM-A8.


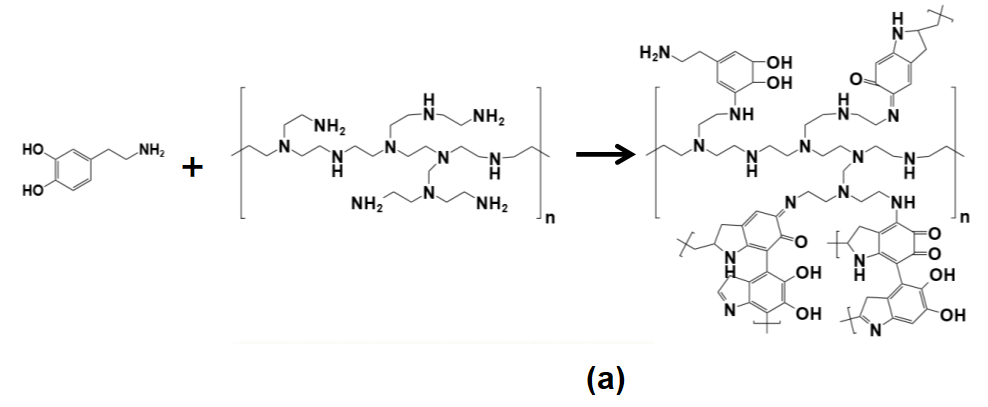


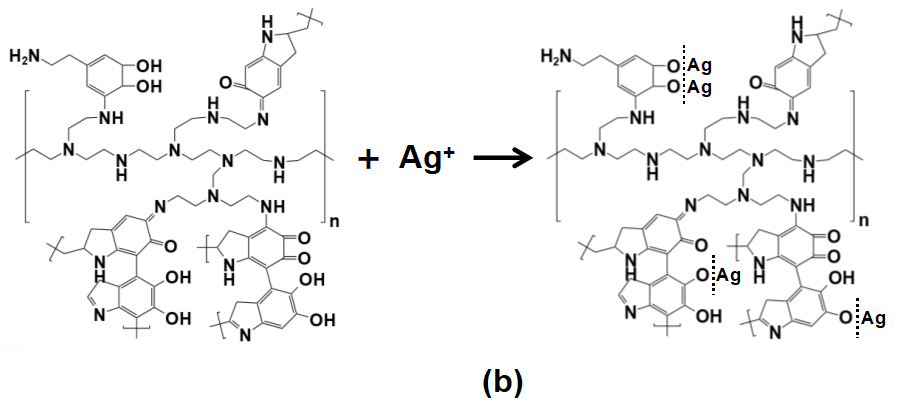


**Figure S8.** (a) Reaction mechanism between the PEI and DA. (b) Assisted deposition mechanism of the Ag nanoparticles.


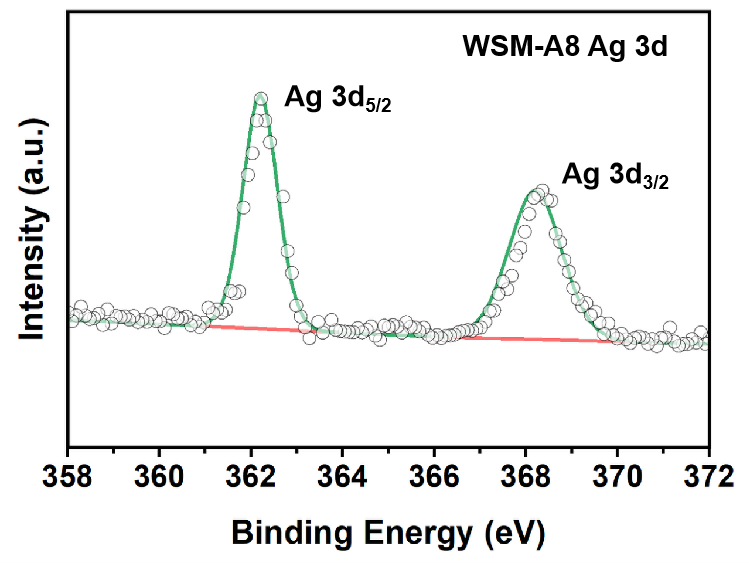


**Figure S9.** XPS survey spectrum for Ag 3d of WSM-A8.


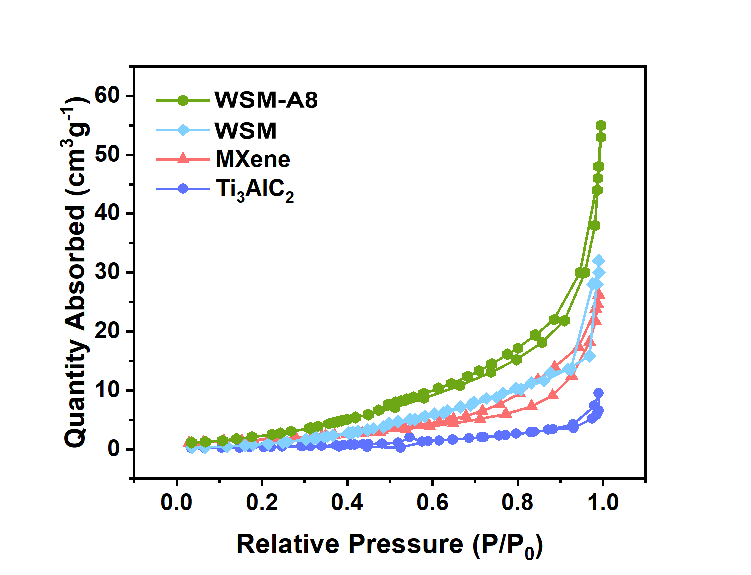


**Figure S10.** Nitrogen sorption isotherms of the Ti_3_AlC_2_ MAX phase, MXene nanosheets, WSM, and WSM-A8.


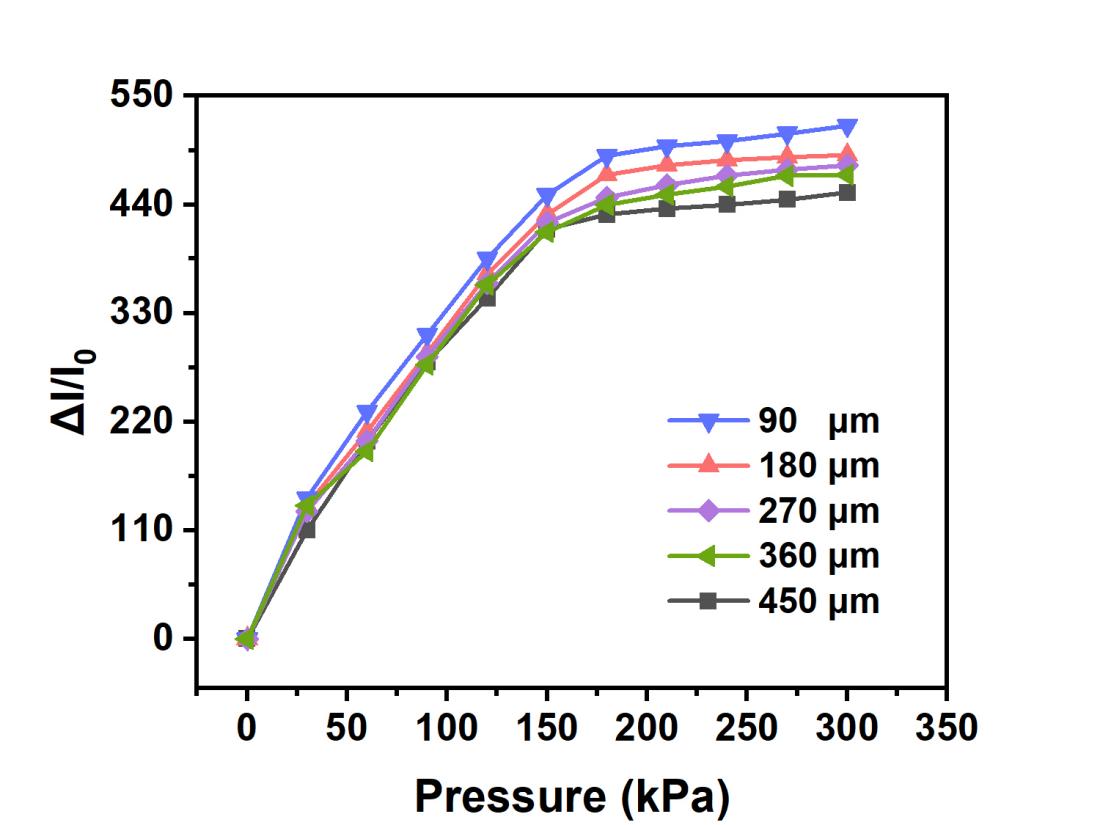


**Figure S1****1.** Response intensity of WSM-A8 pressure sensor with different thickness of pressure sensitive layer.


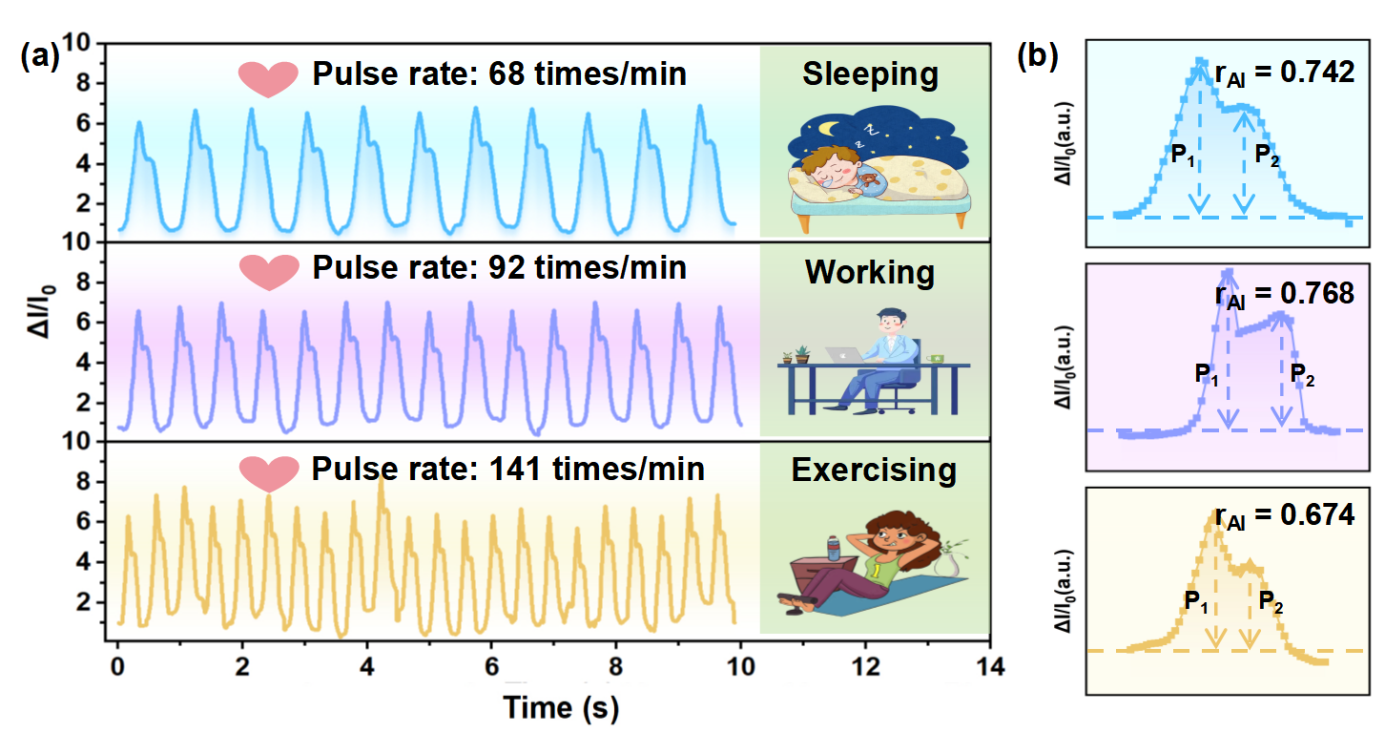


**Figure S12.** Real-time monitoring of a pulse rate and b corresponding magnified waveform of the pulse rate.

As shown in Fig. S14, the pulse rates for the volunteers are calculated to be 68, 92, and 141 times/min during the periods of sleeping, working, and exercising, respectively, which is accordant with the normal level of healthy adults. Meanwhile, based on the magnified waveform of the pulse rate, the r_AI_ for the volunteers are calculated to be 0.742, 0.768, and 0.674 during the periods of sleeping, working, and exercising, respectively, within the normal reference range of the healthy adults.


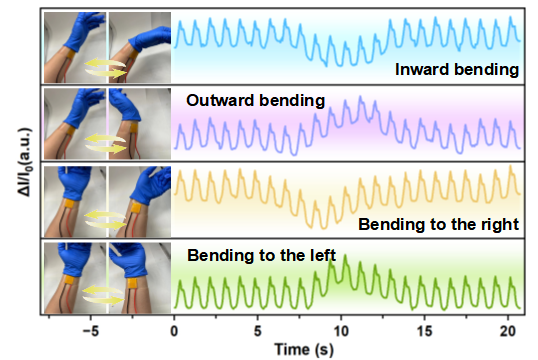


**Figure S13.** Real-time monitoring of pulse signals during the wrist bending with a preload of 5 kPa.

In the process of the pulse rate monitoring with a preload pressure of 5 kPa, wrist bending inward and rightward induce a negative interference to the response signals, while wrist bending outward and leftward induce a positive interference to the response signals.


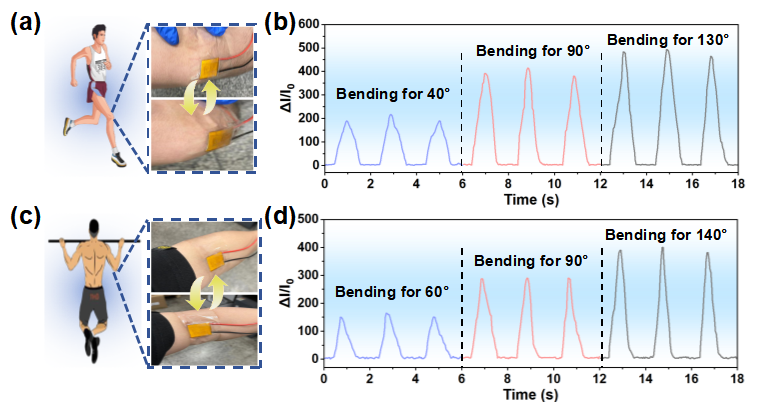


**Figure S14.** (a) Schematic diagram and (b) response signals for the monitoring of the knee bending. (c) Schematic diagram and (d) response signals for the monitoring of the elbow bending.

In Fig. S16a and Fig. S16c, with a WSM-A8 pressure sensor pasted on the knee and elbow, the amplitudes of the knee bending and elbow bending can be effectively monitored through the pressure generated by the deforming of the muscles. Under three various bending angles of the knee and elbow, the response intensity of the WSM-A8 pressure sensor elevates with the increase of the bending angles, which is beneficial for the adjustment of exercise posture and prevention of diseases (Fig. S16b and Fig. S16d).


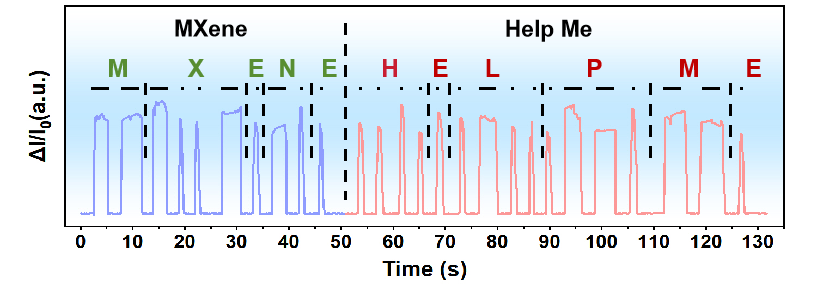


**Figure S15.** Morse code recognition by the WSM-A8 pressure sensor.

Due to the excellent pressure sensing properties, the information can be conveyed by the dots and dashes of Morse code, which is represented by the response signals of long pressing and tapping to the WSM-A8 pressure sensor. As illustrated in Fig. S17, response signals corresponding to the Morse code of “MXene” and “Help Me” can be produced by touching the pressure sensor.


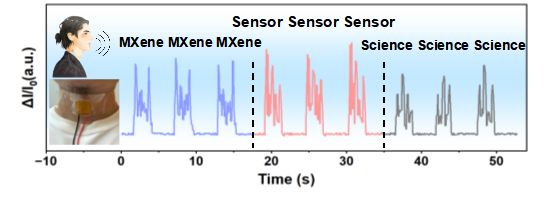


**Figure S16.** Speech recognition by the WSM-A8 pressure sensor.

The signal curves for the words of “MXene”, “Sensor”, and “Science” possess three various unique waveforms, respectively, demonstrating the exceptional speech recognition ability and accurate biological information recording ability of the WSM-A8 pressure sensor. Thus, the WSM-A8 pressure sensor is constructive to enhance the development of the encryption and transmission of security information.


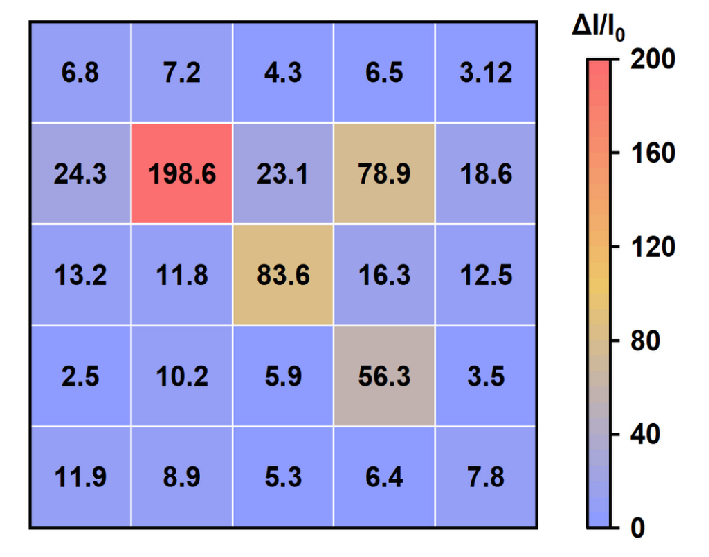


**Figure S17.** Response signal of the WSM-A8 sensor array for the weights.


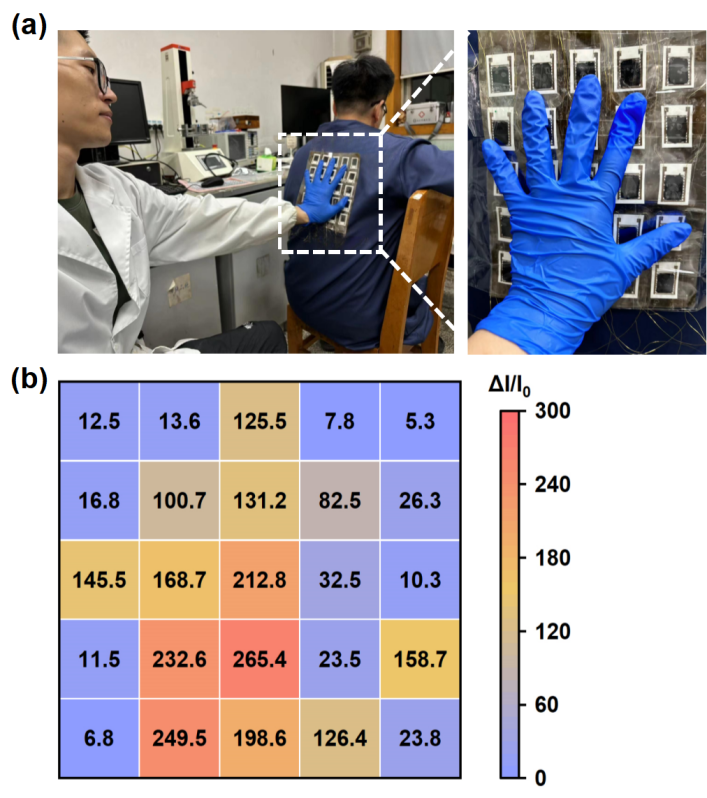


**Figure S18.** (a) Schematic diagram for the pressure monitoring of WSM-A8 sensor array attached to the human back. (b) Response signal of the WSM-A8 sensor array for the palm pressing.


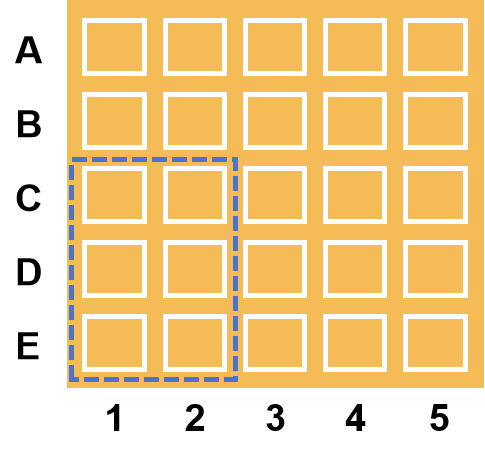


**Figure S19.** Coordinate setting of the six adjacent pixels for the recognition of handwritten Greek letters.


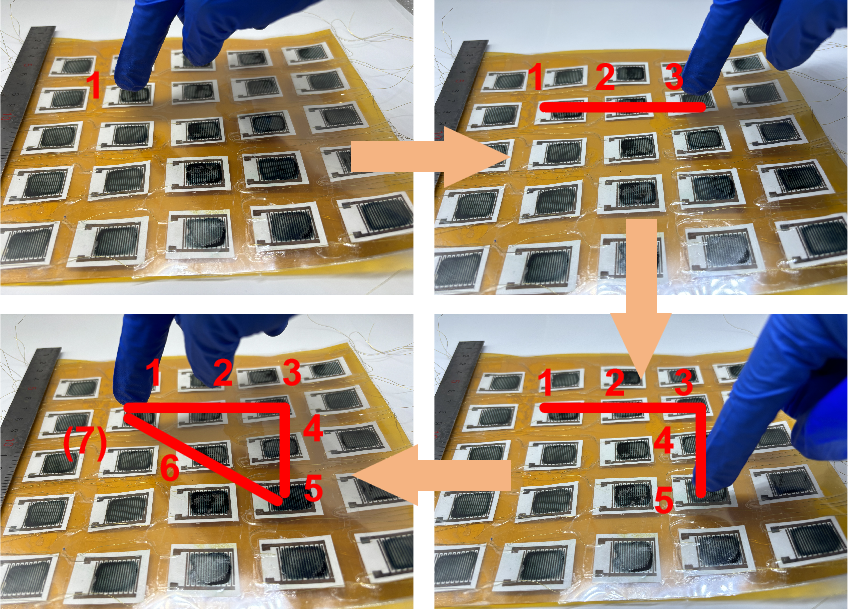


**Figure S20.** Photographs of the finger movements on the sensor array.


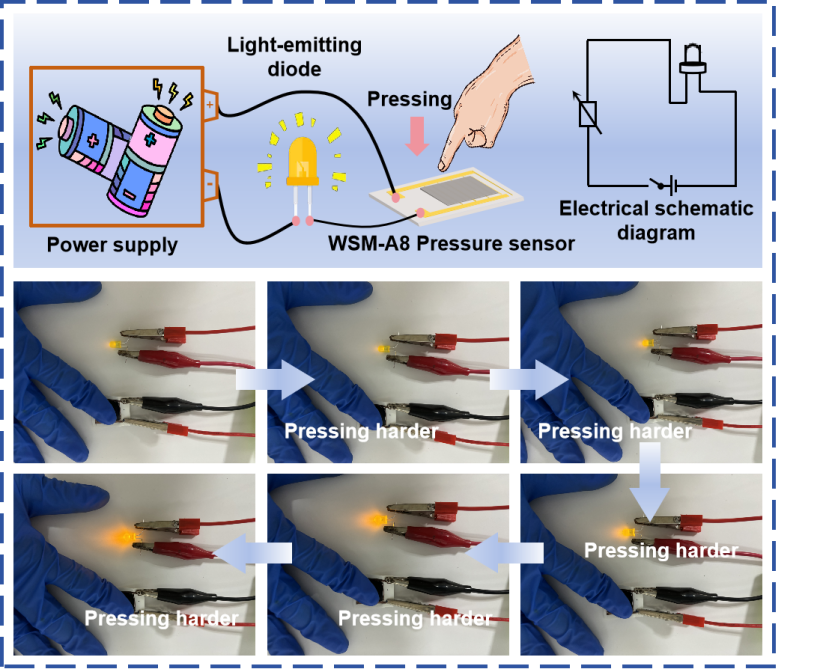


**Figure S21.** Luminance control of the LED light with WSM-A8 pressure sensor.


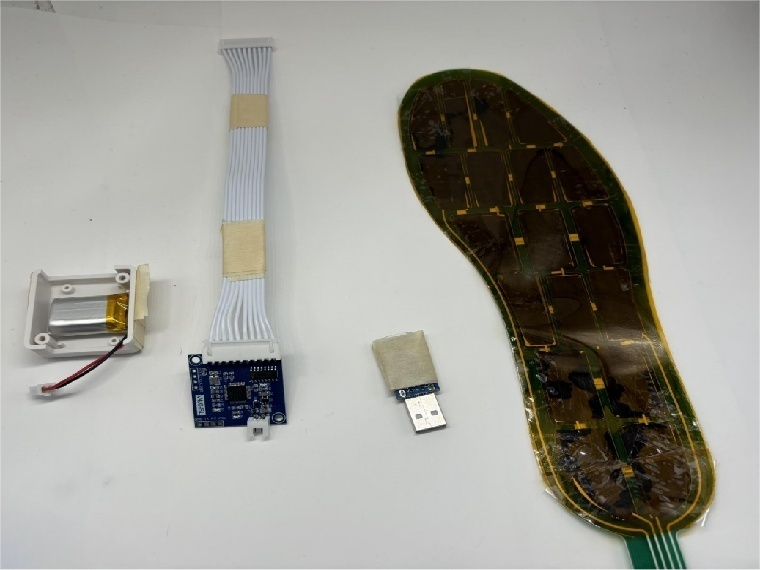


**Figure S22.** Photograph of the wireless WSM-A8 plantar pressure monitor.


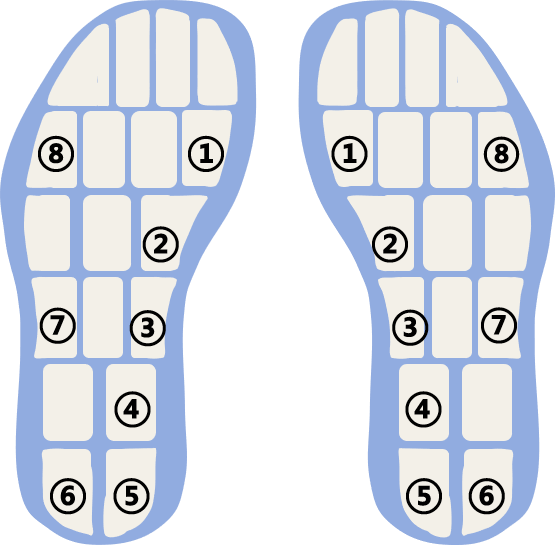


**Figure S23.** The 8 selected representative sensors of the plantar.


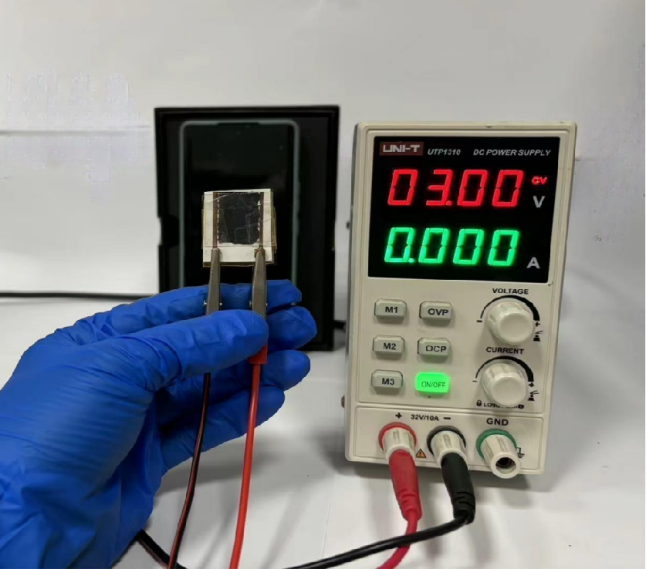


**Figure S24.** Photograph of WSM-A8 pressure sensor with a constant supply voltage of 3 V.


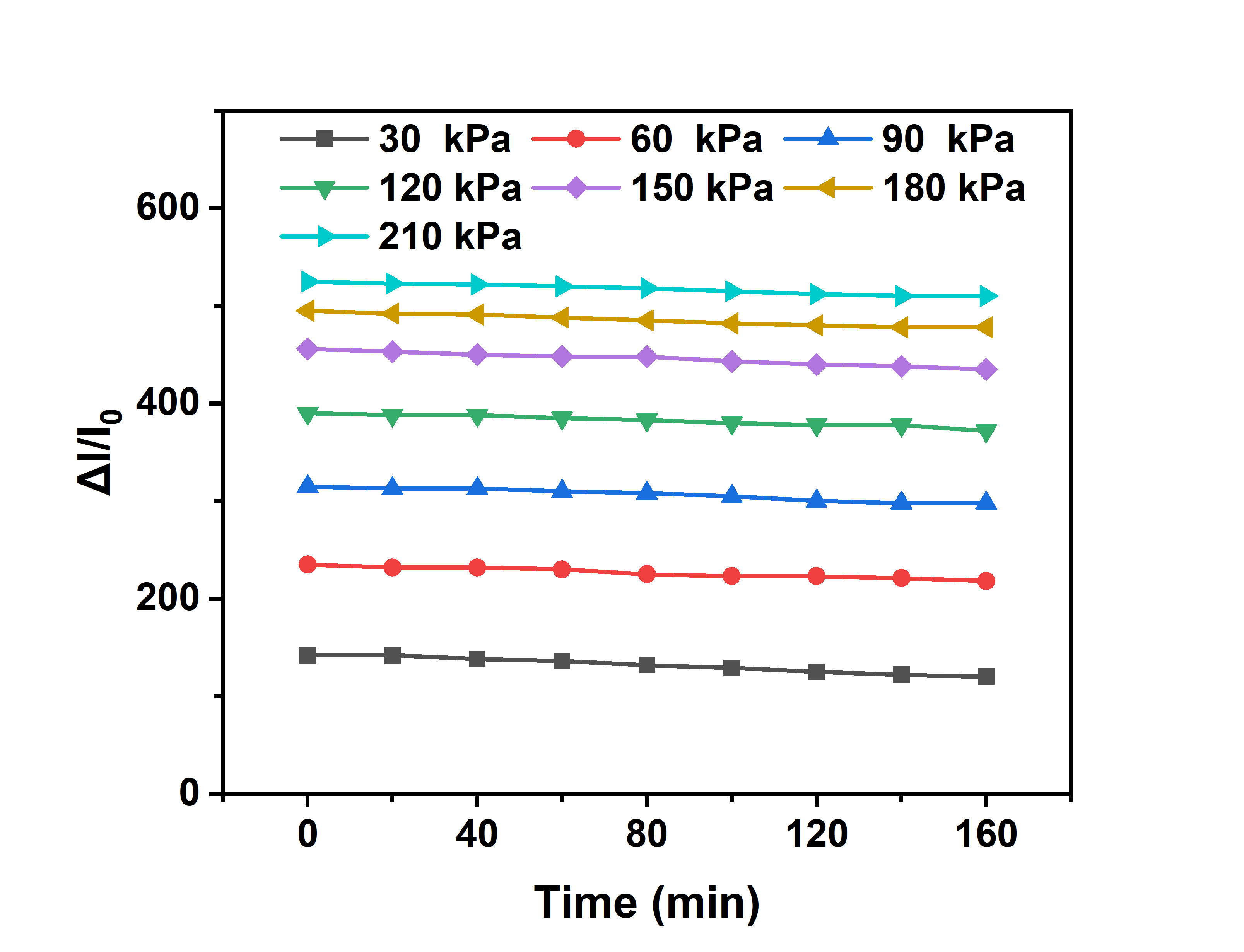


**Figure S25.** Response stability of WSM-A8 pressure sensor on the heating platform with an initial temperature of 130 ℃.


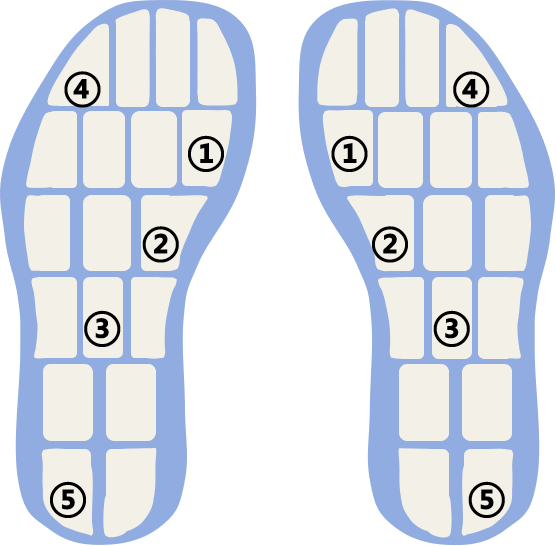


**Figure S26.** Schematic of the 5 selected positions in the wireless WSM-A8 plantar pressure monitor.


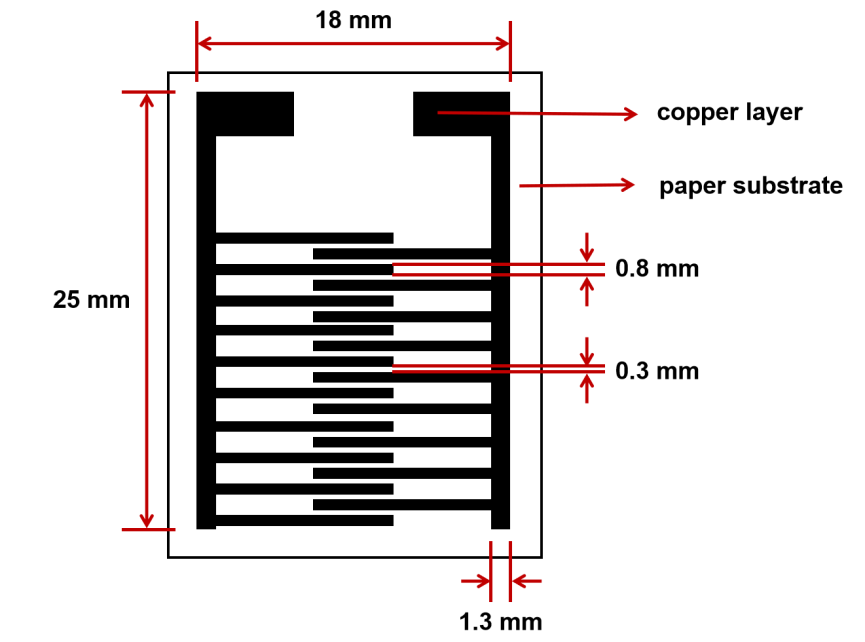


**Figure S27.** The schematic diagram of the copper electrode with paper-based substrate.


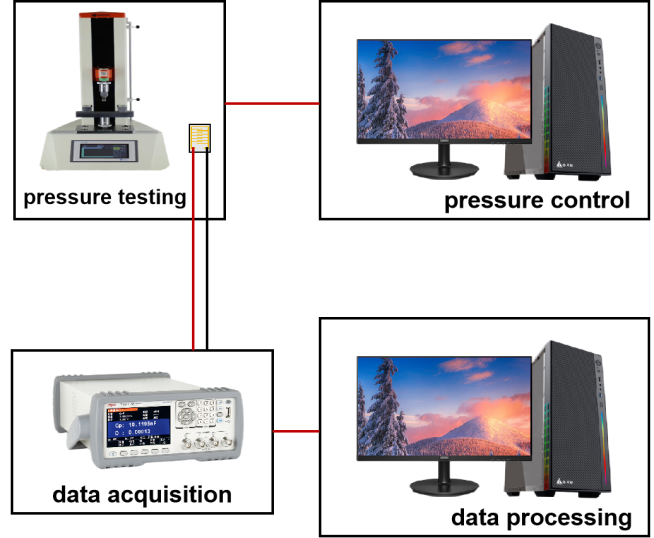


**Figure S28.** Schematic illustration of the experimental facility for pressure sensing test of the pressure sensor.

**Table S1** Comparison of the monitoring sensitivities and sensing ranges of the reported pressure sensors with this work.

| **Material** | **Sensitivity (kPa^-1^)** | | **Sensing range (kPa)** | **Reference** |
| --- | --- | --- | --- | --- |
| PAAm/NaCl | 0.24  1.5  0.13 | 0-70  70-150  150-330 | | [1] |
| PDMS/SWNTs | 0.7 | 0-25 | | [2] |
| PVP/MXene | 1.25  0.88 | 0.13-98  98-294 | | [3] |
| PDMS/PPy/PVDF | 6.23 | 0-100 | | [4] |
| Ecoflex/CNT | 3.13  1.65  1.16  0.68  0.43 | 0-1  1-5  5-10  10-30  30-50 | | [5] |
| MXene/ANF | 0.4986  0.0559 | 0-1  1-10 | | [6] |
| MXene/CCS | 3.84  0.18 | 0-12.4  12.4-809 | | [7] |
| MXene/CNC/TPU | 1.28  0.93  0.76  0.21 | 0-2  2-10  10-25  25-30 | | [8] |
| Cu/PU | 0.0024  0.0007 | 0-10  14-50 | | [9] |
| CNTs/PU | 1.28  0.93  0.76  0.21 | 0-2  2-10  10-25  25-30 | | [10] |
| TPU/PVDF-HFP | 0.781  0.00115 | 0-12  12-30 | | [11] |
| AgNPs/SBS | 0.21  0.064 | 0-2  2-10 | | [12] |
| WSM-A8 | 3.04  1.52  0.365 | 0-120  120-165  165-300 | | This work |

**Table S2** Comparison of the response/recovery time and sensing ranges of the typical MXene-based pressure sensors with this work.

| **Material** | **T_res_+T_rec_** | **Sensing range (kPa)** | **Reference** |
| --- | --- | --- | --- |
| MXene/NF | 550 ms | 0-150 | [13] |
| MXene/SnO_2_ | 340.5 ms | 0-58.78 | [14] |
| MXene/PET | 840 ms | 0-120 | [15] |
| MXene/Nanofibers | 418 ms | 0-48 | [16] |
| MXene/Collagen-fiber | 450 ms | 0-3 | [17] |
| MLNM-MXene | 154 ms | 0-117.5 | [18] |
| MXene-TPU | 170 ms | 0-150 | [19] |
| MXene AP | 800 ms | 0-290 | [20] |
| MXene/RGO | 30 s | 0-287 | [21] |
| MXene/BC/PPy | 185 ms | 0-3 | [22] |
| MXene/CNF | 81 ms | 0-2 | [23] |
| WSM-A8 | 75 ms | 0-300 | This Work |

**Finite element analysis:**

The finite element analysis was performed using the commercial software COMSOL. Based on empirical measurements, the WSM-A8 were simulated as incompressible neo-Hookean materials with Young’s moduli of 0.5 GPa. The MXene nanosheets was simply treated as hard plates and compressed downward. All contact points were assumed to be frictionless and penetration-free. The contact points of the integrated model were assumed to be one piece.

**Microscopic first-principles calculations:**

The density functional theory (DFT) calculations were conducted in the Vienna Ab-initio simulation package (VASP) on the basis of the plane-wave basis sets with the projector augmented-wave method. The exchange-correlation potential was processed by utilizing a generalized gradient approximation (GGA) with the Perdew-Burke-Ernzerhof (PBE) parametrization. The van der Waals correction of Grimme’s DFT-D3 model was also adopted. The energy cutoff was set to be 520 eV. The Brillouin-zone integration was sampled with a Γ-centered Monkhorst-Pack mesh of 13×13×3. The structures were relaxed until the maximum force on each atom was less than 0.01 eV/Å, and the energy convergent standard was 10^-5^ eV.

**Machine learning for classification**

Data preprocessing: The database used in this work was acquired by testing the input information of standing postures and Greek letters 1000 times, respectively. To facilitate effective training within the convolutional neural network framework, raw 2D sample matrices were subjected to row-major vectorization. Each vectorized sample matrix was subsequently flattened to preserve the feature information of the data, enabling the model to operate efficiently.

Division of training set, test set, and validation set: The input data set was divided into training set and test set in the ratio of 4:1. During the training process of the model, 20% of the data in the training set was drawn out as the validation set for the validation of the model.

Convolutional neural network structure: To mitigate overfitting risks arising from excessive co-adaptation of neurons and enhance the generalization capability of the model, a dropout layer with a hyperparameter rate of 0.5 was strategically integrated into the convolutional neural network architecture. This regularization mechanism randomly deactivates 50% of neuronal activations during forward/backward propagation, enforcing distributed feature learning and reducing reliance on specific local patterns effectively. The discarded information possessed the characteristic of randomness, simulating ensemble training over exponentially many sub-networks while maintaining computational efficiency during inference through weight scaling.

**References**

1. Z. Q. Shen, X. Y. Zhu, C. Majidi, G. Y. Gu, Cutaneous ionogel mechanoreceptors for soft machines, physiological sensing, and amputee prostheses. Adv. Mater. **2021**, 33, 2102069.
2. G. Y. Bae, J. T. Han, G. Lee, S. Lee, S. W. Kim, S. Park, J. Kwon, S. Jung, K. Cho, Pressure/temperature sensing bimodal electronic skin with stimulus discriminability and linear sensitivity. Adv. Mater. **2018**, 30, 1803388.
3. J. Tao, M. Dong, L. Li, C. F. Wang, J. Li, Y. Liu, R. R. Bao, C. F. Pan, Real-time pressure mapping smart insole system based on a controllable vertical pore dielectric layer. Microsyst. Nanoeng. **2020**, 6, 62.
4. Y. Zheng, T. Lin, N. Zhao, C. X. Huang, W. Chen, G. Xue, Y. Wang, C. Teng, X. L. Wang, D. S. Zhou, Highly sensitive electronic skin with a linear response based on the strategy of controlling the contact area. Nano Energy **2021**, 85, 106013.
5. T. Hu, B. Sheng, A highly sensitive strain sensor with wide linear sensing range prepared on a hybrid-structured CNT/ecoflex film via local regulation of strain distribution. ACS Appl. Mater. Interfaces **2024**, 16, 21061-21072.
6. H. Jiang, M. Qin, C. Zhang, Z. W. Weng, J. B. Zhang, X. Weng, Mixed-dimensional MXene nanocomposites/aramid nanofibers-based flexible pressure and strain sensor for electronic skin. ACS Appl. Electron. Mater. **2023**, 5, 2276-2287.
7. Z. P. Yang, H. Q. Li, S. F. Zhang, X. J. Lai, X. R. Zeng, Superhydrophobic MXene@carboxylated carbon nanotubes/carboxymethyl chitosan aerogel for piezoresistive pressure sensor. Chem. Eng. J. **2021**, 425, 130462.
8. Q. M. Li, R. Yin, D. B. Zhang, H. Liu, X. Y. Chen, Y. J. Zheng, Z. H. Guo, C. T. Liu, C. Y. Shen, Flexible conductive MXene/cellulose nanocrystal coated nonwoven fabrics for tunable wearable strain/pressure sensors. J. Mater. Chem. A **2020**, 8, 21131-21141.
9. M. Chen, J. Y. Ouyang, A. J. Jian, J. Liu, P. Li, Y. X. Hao, Y. C. Gong, J. Y. Hu, J. Zhou, R. Wang, J. X. Wang, L. Hu, Y. W. Wang, J. Ouyang, J. Zhang, C. Hou, L. Wei, H. M. Zhou, D. Y. Zhang, G. M. Tao, Imperceptible, designable, and scalable braided electronic cord. Nat. Commun. **2022**, 13, 7097.
10. X. Y. Qu, J. Li, Z. L. Han, Q. Q. Liang, Z. Zhou, R. M. Xie, H. P. Wang, S. Y. Chen, Highly sensitive fiber pressure sensors over a wide pressure range enabled by pesistive-capacitive hybrid response. ACS Nano **2023**, 17, 14904-14915.
11. P. C. Uzabakiriho, M. Wang, C. Ma, G. Zhao, Stretchable, breathable, and highly sensitive capacitive and self-powered electronic skin based on core-shell nanofibers. Nanoscale **2022**, 14, 6600-6611.
12. J. Lee, H. Kwon, J. Seo, S. Shin, J. H. Koo, C. Pang, S. Son, J. H. Kim, Y. H. Jang, D. E. Kim, T. Lee, Conductive fiber-based ultrasensitive textile pressure sensor for wearable electronics. Adv. Mater. **2015**, 27, 2433-2439.
13. Q. H. Yu, C. L. Su, S. Y. Bi, Y. L. Huang, J. N. Li, H. Q. Shao, J. H. Jiang, N. L. Chen, Ti_3_C_2_T_X_@nonwoven fabric composite: promising MXene-coated fabric for wearable piezoresistive pressure sensors. ACS Appl. Mater. Interfaces **2022**, 14, 9632-9643.
14. A. Mahajan, S. Gasso, Self-powered wearable gas sensors based on L-ascorbate-treated MXene nanosheets and SnO_2_ nanofibers. ACS Appl. Nano Mater. **2023**, 6, 6678-6692.
15. H. L. Liu, Q. Zhang, N. Yang, X. Z. Jiang, F. Wang, X. Yan, X. A. Zhang, Y. Zhao, T. L. Cheng, Ti_3_C_2_T_X_ MXene paper-based wearable and degradable pressure sensor for human motion detection and encrypted information transmission. ACS Appl. Mater. Interfaces **2023**, 15, 44554-44562.
16. L. Wang, M. Y. Zhang, B. Yang, J. J. Tan, X. Y. Ding, Highly compressible, thermally stable, light-weight, and robust aramid nanofibers/Ti_3_AlC_2_ MXene composite aerogel for sensitive pressure sensor. ACS Nano **2020**, 14, 10633-10647.
17. W. B. Zhang, Z. Y. Pan, J. Z. Ma, L. F. Wei, Z. Chen, O. Wang, Degradable cross-linked collagen fiber/MXene composite aerogels as a high-performing sensitive pressure sensor. ACS Sustain. Chem. Eng. **2022**, 10, 1408-1418.
18. B. Q. Jia, Z. K. Li, T. F. Zheng, J. X. Wang, Z. J. Zhao, L. B. Zhao, B. Wang, J. J. Lu, K. Zhao, G. X. Luo, M. Li, Q. J. Lin, Z. D. Jiang, Highly-sensitive, broad-range, and highly-dynamic MXene pressure sensors with multi-level nano-microstructures for healthcare and soft robots applications. Chem. Eng. J. **2024**, 485, 149750.
19. Z. Zheng, X. Fang, Y. F. Pan, S. Y. Song, H. Xue, J. Li, Y. Li, J. Li, A liquid metal-embedded 3D interconnected-porous TPU/MXene composite with improved capacitive sensitivity and pressure detection range. RSC Adv. **2024**, 14, 15730-15741.
20. D. J. Yao, Z. H. Tang, L. Zhang, Z. G. Liu, Q. J. Sun, S. C. Hu, Q. X. Liu, X. G. Tang, J. Y. Ouyang, A highly sensitive, foldable and wearable pressure sensor based on MXene-coated airlaid paper for electronic skin. J. Mater. Chem. C **2021**, 228, 111814.
21. H. Y. Qiao, W. J. Qin, J. J. Chen, L. Feng, C. S. Gu, M. Yang, Z. H. Tian, J. X. Chen, X. X. Li, Y. L. Wang, S. G. Yin, AuCu decorated MXene/RGO aerogels towards wearable thermal management and pressure sensing applications. Mater. Des. **2023**, 228, 111814.
22. W. F. Zheng, Y. Yang, L. L. Fan, D. Z. Ye, W. L. Xu, J. Xu, Ultralight PPy@PVA/BC/MXene composite aerogels for high-performance supercapacitor eltrodes and pressure sensors. Appl. Surf. Sci. **2023**, 624, 157138.
23. L. Y. Qin, D. Z. Yang, M. Zhang, T. Y. Zhao, Z. Luo, Z. Z. Yu, Superelastic and ultralight electrospun carbon nanofiber/MXene hybrid aerogels with anisotropic microchannels for pressure sensing and energy storage. J. Colloid Interface Sci. **2021**, 589, 264-274.
